# Morphogenetic development of trochlear groove and thigh muscles from embryo to fetus in humans

**DOI:** 10.1371/journal.pone.0339167

**Published:** 2026-02-02

**Authors:** Aoi Ishikawa, Momoko Nagai-Tanima, Kanon Ishida, Hirohiko Imai, Akio Yoneyama, Shigehito Yamada, Hiroki Otani, Tomoki Aoyama, Tetsuya Takakuwa

**Affiliations:** 1 Human Health Sciences, Graduate School of Medicine, Kyoto University, Kyoto, Japan; 2 Department of Systems Science, Kyoto University Graduate School of Informatics, Kyoto, Japan; 3 SAGA Light Source, Saga, Japan; 4 Congenital Anomaly Research Center, Graduate School of Medicine, Kyoto University, Kyoto, Japan; 5 Department of Developmental Biology, Shimane University Faculty of Medicine, Izumo, Shimane, Japan; Tokai University, School of Medicine, JAPAN

## Abstract

Mechanical forces caused by fetal movements are essential for normal musculoskeletal development. However, the relationship between skeletal development and knee joint motion in utero remains unclear. We aimed to clarify the development of lower limb musculoskeletal structures during the embryonic and early fetal stages from a kinematic perspective and to compare muscle and skeletal developmental processes. We analyzed 29 human embryonic and fetal specimens. Using phase-contrast X-ray computed tomography and magnetic resonance imaging, we analyzed the morphogenesis of the knee joint components, trochlear groove angle, and hypothetical joint motion of the thigh muscles in three dimensions. To analyze chronological morphogenesis, Procrustes analysis was performed, and Principal component analysis and linear discriminant analysis were subsequently conducted using the Procrustes coordinates. The trochlear groove angle on the plane vertical to the femoral axis decreased from approximately 160° to 130° until 120 mm Crown-rump length (CRL) and remained constant until 185 mm CRL. All hypothetical joint motions increased slowly between 30 and 100 mm CRL, whereas rapidly after approximately 100 mm CRL. The protrusions of the lateral femoral condyle became more pronounced from fetal stage as they grew. The timing of the onset of fetal movement and the increase in muscle mass and joint motion during early pregnancy were consistent with those of previous studies, and the timing of the angle stabilization occurred almost simultaneously with the rapid increase in femoral muscle mass and hypothesized joint motion. It suggested that stabilization of joint morphology enables smooth joint motion, which leads to an increase in muscle mass and joint motion.

## Introduction

The knee joint is a complex three-dimensional structure composed of multiple tissues, such as bone, cartilage, and ligaments. The development of the knee structure has been clarified through various studies using mammal models. Such a structure develops through both a determined program and passive factors [1]. Several studies have reported that mechanical forces from fetal movements stimulate the fetal skeleton and are essential for normal musculoskeletal development [2–4], while mechanosensitive gene expression is involved in joint formation [5–8]. In humans, biomechanical stimulation by uterine wall kicking influences skeletal and joint formations in lower leg skeletal development [9]. Muscle contraction affects the structure and motion of the knee [10,11]. Joint motion, a rotational movement around the joint center, is generated by muscle contraction characterized by both muscle force and moment arm, which together determine the magnitude of rotation and are quantified as joint torque in the mature human body [12,13]. Besides, the longer the moment arm, the greater the rotational force on the joint [14]. Therefore, during development, the rotation force on joints might be determined by identifying the growth-associated increase in muscle force and moment arm.

In human skeletal development, the femur first appears as mesenchymal condensation between Carnegie stage (CS) 16 and CS17. CS is a standardized system comprising 23 stages that provide a unified developmental chronology of the vertebrate embryonic stage [15]. Chondrification occurs between CS17 and CS18 and subsequently proceeds to endochondral ossification between CS22 and CS23 [4,16,17]. Cells form limb muscle masses around the bone primordium and separate into individual skeletal muscles, with lower limb development occurring a few days after upper limb development [18]. Muscle maturation proceeds from proximal to distal muscles, with the main muscles resembling those of adults by the end of the embryonic period or CS23 in humans [19]. In recent years, magnetic resonance (MR) imaging and phase-contrast X-ray computed tomography (CT) have enabled the three-dimensional (3D) analysis of the internal structure of specimens, such as human embryos. Several musculoskeletal structural studies have been conducted using human embryos [20–22]. The 3D structural knowledge from human embryos and fetuses has been essential for understanding the normal developmental process and development-related diseases.

The knee consists of two joints, the patellofemoral and tibiofemoral joints. Trochlear dysplasia significantly affects the development of patellofemoral instability. In clinical practice, patellofemoral instability results from dysplasia of the trochlear groove in the distal femoral epiphysis. Patients with patellar instability can experience debilitating pain, limitations in basic function, and long-term arthritis [23]. Trochlear dysplasia is defined as a trochlear groove angle greater than 145° [23,24] that may result from a decreased lateral trochlear slope of the lateral trochlear facet or a decreased central trochlear depth [23–25]. Several studies have investigated the trochlear groove development process from fetus to adulthood [17,26–28]. Bony trochlear groove angle in fetuses has been reported to resemble that in adults [27]. In healthy individuals, the cartilaginous trochlear slope develops properly and is nearly in adult form at birth [26], while during childhood (3–16 years), the trochlear groove angle remains constant, with the lateral trochlear groove being higher than that of the medial and continuing to grow across all ages [17]. However, the chronological changes from embryonic to fetal stages remain unclear. In addition, no studies have analyzed the relationship between trochlear groove formation and knee joint motion, including muscle development, in utero.

This study aimed to clarify the development of lower limb musculoskeletal structures during the embryonic and early fetal stages from a kinematic perspective and to compare muscle and skeletal developmental processes using reconstructed 3D images. In particular, we focused on the morphogenesis of the femoral trochlea of the knee joint and hypothesized that trochlear formation progresses in association with periarticular muscle development and relative joint movements such as flexion and extension, and internal and external rotation during intrauterine development.

## Materials and methods

### Human embryonic and fetal specimens

Twenty-nine human embryonic and fetal specimens (eight embryonic CS18–CS23 specimens: crown-rump length (CRL) range of 11.4–33.5 mm, and 21 fetal specimens: CRL range of 37.2–185 mm) from the Kyoto Collection at the Congenital Anomaly Research Center of Kyoto University, Japan [29] and Shimane University were used (Table 1). These specimen’s data were accessed in 1/4/2020–31/6/2021. The right lower limb was used for image analysis. Several additional serial histological sections of human embryos from the Kyoto Collection of Human Embryos (CS18–CS23) were used to confirm the start of trochlear groove formation. Most specimens stored in the Kyoto Collection are from pregnancies that were terminated for socioeconomic reasons under the Maternity Protection Law of Japan. Samples were collected between 1963 and 1995 following the relevant regulations of these periods. Written informed consent was not required at that time; however, verbal informed consent for depositing the specimens was provided by parents, as documented in their medical records. All samples were anonymized and deidentified. The aborted fetal specimens were preserved in 10% formalin. Measurements and examinations were performed, and specimens were staged using the criteria proposed by O’Rahilly and Müller [15,19,30]. For histological observation, each specimen was sliced into 10-μm-thick slices horizontally, coronally, or sagittally, stained with hematoxylin and eosin, and observed under an Olympus VS120 virtual slide system (Olympus, Tokyo, Japan) at low and high magnifications.

**Table 1:** List of the specimens.

Specimen ID	Storage	Carnegie stage	Image type	GA (d)	CRL (mm)	Sex	Femur length (mm)
17746	Kyoto Collection	18	CT	52	11.4	–	1.21
16127	Kyoto Collection	19	CT	59	14.8	–	1.35
22171	Kyoto Collection	20	CT	65	16.5	–	1.84
32721	Kyoto Collection	21	CT	65	21.0	–	2.49
28066	Kyoto Collection	21	CT	80	22.6	–	2.50
35233	Kyoto Collection	22	7T MRI	56	21.2	–	2.98
25796	Kyoto Collection	23	7T MRI	84	26.8	–	4.50
92310	Kyoto Collection	23	7T MRI	71	33.5	–	5.52
52002	Kyoto Collection	Fetus	7T MRI	104	37.2	–	7.19
33563	Kyoto Collection	Fetus	7T MRI	117	43.5	M	10.77
51128	Kyoto Collection	Fetus	7T MRI	82	52.0	F	11.66
33087	Shimane Univ.	Fetus	7T MRI	90	58.7	F	14.79
51272	Kyoto Collection	Fetus	7T MRI	99	62.0	M	14.19
92240	Shimane Univ.	Fetus	7T MRI	93	70.5	F	18.70
92949	Kyoto Collection	Fetus	7T MRI	63	84.5	F	24.90
37304	Shimane Univ.	Fetus	7T MRI	101	87.5	F	20.80
53520	Shimane Univ.	Fetus	7T MRI	102	97.0	M	24.03
70323	Kyoto Collection	Fetus	7T MRI	N.D.	103.0	N.D.	24.89
91915	Shimane Univ.	Fetus	7T MRI	109	112.0	M	32.14
91892	Shimane Univ.	Fetus	7T MRI	136	117.0	M	35.90
53273	Shimane Univ.	Fetus	3T MRI	120	122.7	M	25.36
37626	Kyoto Collection	Fetus	7T MRI	136	128.1	M	29.73
37866	Shimane Univ.	Fetus	7T MRI	131	129.3	M	31.43
53178	Shimane Univ.	Fetus	3T MRI	123	147.0	M	41.51
91517	Kyoto Collection	Fetus	3T MRI	136	148.0	F	42.63
53571	Kyoto Collection	Fetus	3T MRI	138	163.0	M	40.55
53444	Kyoto Collection	Fetus	3T MRI	133	163.0	F	41.06
53503	Kyoto Collection	Fetus	3T MRI	N.D.	170.0	M	46.90
53467	Kyoto Collection	Fetus	3T MRI	N.D.	185.0	F	47.92

N.D. means no data.

This study was approved by the Ethics Committee of the Kyoto University Faculty and Graduate School of Medicine (E986, R0316, and R2224).

### Image acquisition

MR images were acquired using 3T (MAGNETOM Prisma; Siemens Healthineers, Erlangen, Germany) and 7T (BioSpec 70/20 USR; Bruker BioSpin MRI GmbH, Ettlingen, Germany) MR systems equipped with ¹H quadrature transmit-receive volume coils of 35 and 72 mm in diameter (T9988 and T9562; Bruker BioSpin MRI GmbH).

The image acquisition parameters for 3D CT have been described previously [31]. Briefly, the specimens were visualized using a phase-contrast imaging system fitted with a crystal X-ray interferometer [32]. The system was set up at the vertical wiggler beamline (PF BL-14C) of the Photon Factory at Inter-University Research Institute Corporation High Energy Accelerator Research Organization (Tsukuba, Japan). The white synchrotron radiation emitted from the wiggler was monochromated using a Si(220) double-crystal monochromator, horizontally magnified through an asymmetric crystal, and entered into the imaging system. The generated interference patterns were detected using a large-area X-ray imager comprising a 30-μm scintillator, a relay lens system, and a water-cooled charge-coupled device camera (field of view 36 × 36 mm, 2048 × 2048 pixels, 18 × 18 μm each) [33]. The X-ray energy was tuned to 17.8 keV, and an exposure time of 3 s was used to obtain one interference pattern. The average intensity was approximately 300 counts/pixel, allowing high-resolution observations within a reasonable measurement time. The image acquisition method was selected based on the specimen resolution and volume. CT can acquire images with higher resolution than MR images. However, CT cannot be used to acquire images of large-volume specimens. During image acquisition, the specimens were embedded in 1% agarose gel.

### Image analysis

The CT and MR imaging data for the selected specimens were precisely analyzed using serial 2D and reconstructed 3D images. CT was used for 3D reconstructed images of the embryonic period, whereas 7T and 3T MR images were used for 3D reconstructed images from CS22 of the embryonic to the fetal period (Table 1). The images of the knee joint structures, including the femur, tibia, patella, hamstring muscle (semitendinosus, semimembranosus, long and short head of biceps femoris), and quadriceps femoris muscle (rectus femoris and vastus muscles), were manually segmented, and 3D reconstructed images were generated using the Thermo Scientific^TM^ Amira^TM^ software version 2020.3 (Visage Imaging GmbH, Berlin, Germany). Each slice was manually segmented using the structure of interest. Based on these reconstructed 3D images, femur length was measured thrice, and the average value was calculated as an indicator of normal development. For all morphometric assessment, the evaluation surface and landmarks were confirmed by three of the authors with expertise in musculoskeletal and anatomical sciences. (AI, MN and AT).

### Trochlear groove angle measurements

**Fig 1:**
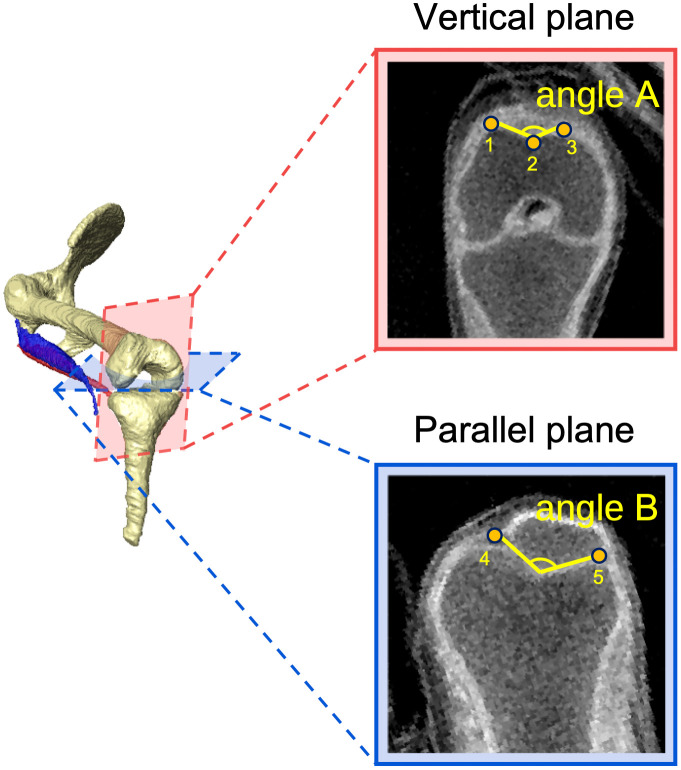
Trochlear groove angle measurements. Magnetic resonance (MR) images of two cross-sections of the knee were used to measure the trochlear groove angle: between the medial condyle of the femur, intercondylar groove, and lateral condyle of the femur. (A) Plane consists of the vertical to the femoral axis, passing through three points: the medial trochlear peak (point #1), trochlear groove (point #2) and lateral trochlear peak (point #3). (B) Plane is parallel to the femoral axis, passing through passing through the peaks of medial (point #4) and lateral (point #5) femoral epicondyles. The patella facet angle on this plane was measured. CRL 52 mm sample image was shown as representing data.

In adult humans, the trochlear groove angle varies depending on the site of femoral evaluation [34] and differences in the patellofemoral joint contact surfaces that occur depending on the degree of knee flexion [35]. To clarify the differences in the patellar groove depending on the evaluation site, trochlear groove angles were assessed on the subchondral bone in two cross-sections, vertical and parallel to the femoral axis (angles A and B, respectively) (Fig 1). Angle A was defined as the patellar position in knee extension, commonly used for assessing the risk of patellar dislocation [26,27], while Angle B was defined as the patellar position in knee flexion [35] to represent the trochlear–patellar relationship during flexion, when the patellar contact area shifts distally along the femoral surface, thereby capturing morphological characteristics across a wider range of knee motion. The femoral axis was defined as the line connecting the proximal end of the lateral surface of the femur and the midpoint of the line between the points of the trochlear groove and the intercondylar notch, which lie on the plane formed by the points of the lateral trochlear apex, medial epicondyle, and lateral epicondyle. For angle A, the plane consisted of three points vertical to the femoral axis: the lateral trochlear peak, trochlear groove and medial trochlear peak. For angle B, the plane was parallel to the femoral axis, passing through the peaks of lateral and medial femoral epicondyles. The patella facet angle on this plane was measured.

### Procrustes analysis of distal femur and morphological landmarks

**Fig 2:**
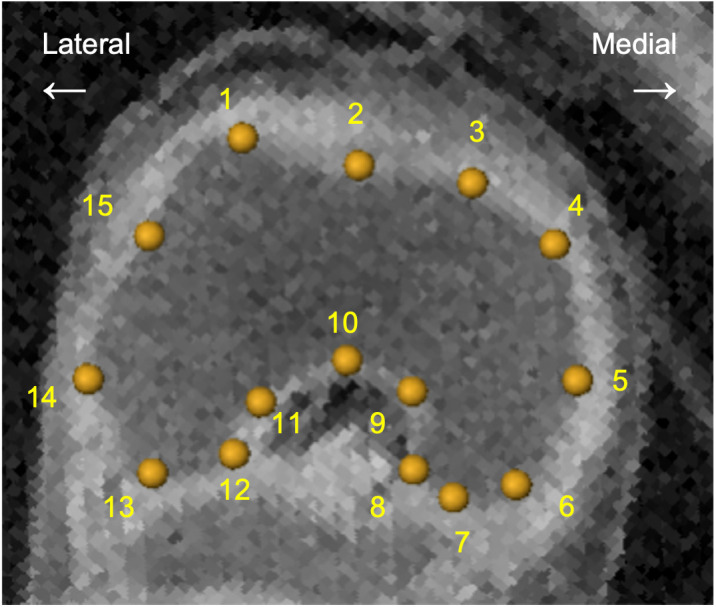
Landmarks of the distal epicondyle coordinates for the distal epiphysis on vertical section for Procrustes analysis. The landmarks of the magnetic resonance (MR) image were 15, including the lateral trochlear peak, medial epicondyle, and lateral epicondyle (No. 1, 5, and 14). Table 2 provides more details regarding these landmarks. CRL 52 mm sample image was shown as representing data.

The morphogenesis of the femoral condyle was examined using Procrustes analysis of the shape of the femoral epiphysis from an axial section of the distal femoral epiphysis. Fifteen landmarks were selected manually and used for the wired models and subjected to Procrustes analysis using software-assisted algorithms (MATLAB R2021a; MathWorks) [20]. The 15 landmarks were morphological points useful for capturing the shape of the distal femur (Fig 2, Table 2) [36]. Procrustes analysis was performed to ensure that the landmark coordinates were translated, scaled, and rotated to the best superimposition. The resultant landmark coordinates are referred to as the Procrustes shape coordinates. Changes in the positions of the landmarks were observed in each Procrustes-shaped coordinate system.

**Table 2:** List of landmarks for Procrustes analysis.

No.	Landmarks	Description (Anatomical landmarks)
1	Lateral trochlear peak	The most anterior point of the lateral trochlear ridge
2	Trochlear groove	The deepest point of the trochlear groove
3	Medial trochlear peak	The most anterior point of the medial trochlear ridge
4	The ridge between the medial trochlear peak and epicondyle	Most prominent bony protrusion between the medial trochlear peak and medial condyle
5	Medial epicondyle	Most prominent bony protrusion over the medial condyle
6	Medial point of the posterior medial condyle	Most medial point of the posterior medial condyle
7	Medial posterior point	Most posterior point on the medial condyle
8	Central point of the posterior medial condyle	Most central point of the posterior medial condyle
9	Medial intercondylar notch	Most medial point of the intercondylar notch
10	Intercondylar notch apex	The most anterior and distal termination of the intercondylar notch
11	Lateral intercondylar notch	Most lateral point of the intercondylar notch
12	Central point of the posterior lateral condyle	Most central point of the posterior lateral condyle
13	Lateral posterior point	Most posterior point on the lateral condyle
14	Lateral epicondyle	Most prominent bony protrusion over the lateral condyle
15	Medial point of the anterior part of the lateral condyle	Medial point between the lateral trochlear peak and lateral epicondyle

### Joint motion measurements

Joint motion involves muscle strength and moment arm. The muscle cross-sectional area (CSA) is proportional to muscle strength [37], while the moment arm refers to the shortest distance between the joint center point and the muscle’s line of action [14]. Joint torque is calculated by multiplying muscle force by moment arm. Inherently, moment arm is affected by the joint flexion angle; however, in this study, the knee joint flexion angles could not be standardized among individuals because the specimens used were formalin-fixed. Therefore, modified moment arm was defined as the line of action (LA), that is, the distance from the knee joint center point (the midpoint of the medial and lateral femoral epicondyles) to the insertion of each muscle in order to eliminate the effect of joint angle in each individual. After completing the 3D reconstruction images of the femur, tibia, the knee joint center point and muscles to be evaluated, the muscle attachment point was set as the center point of the muscle insertion point on the tibia. In addition, muscle strength is influenced by the pennation angle of muscle fascicles. Although reports exist concerning pennation angle in postnatal infants [38], to date no studies have documented pennation angle during the embryonic or fetal stages of development [22,39]. Therefore, in the present study, we employed the mean CSA described below, rather than the physiologic cross-sectional area, which requires consideration of pennation angle. The average CSA was calculated by dividing each muscle volume by the muscle length using the Amira software. The muscle length was defined as a straight line connecting the origin to the insertion of each muscle. To quantitatively assess the joint motion affecting knee joint movement, the hypothetical joint motion was defined as the average CSA of each muscle [mm^2^] × LA [mm] (S1 Fig).

All muscles were reconstructed separately to precisely calculate the muscle volume. For hamstrings, joint motion measurements were conducted using two categories: (1) semitendinosus and semimembranosus and (2) biceps femoris, to simplify the joint motion direction to the knee joint into two directions, internal and external. The total muscle mass of each category and the length of the longer muscle in each category were used as muscle length in the CSA calculations. For evaluating the joint motions in two opposite directions of muscle action, flexion and extension of the knee joint, the sums per muscle action were compared. Regarding muscle length, the attachment sites of the semitendinosus (ST) and semimembranosus (SM) on the tibia were indistinct and could not be clearly differentiated; therefore, they were treated as having a common insertion. In the evaluation of muscle volume, when the muscles were too small to allow accurate calculation of individual volumes (Sample ID 25796, CRL 26.8), ST and SM were evaluated together as a single set, and the long and short heads of the biceps femoris (LHBF and SHBF) were also evaluated together as one set, due to their indistinguishable boundaries. In contrast, samples in which the boundaries between muscles were indistinct (Sample ID 37866, CRL 129.3 mm and Sample ID 53467, CRL 185.0 mm) could not be analyzed and were therefore omitted from muscle evaluation.

### Statistical analysis

For the trochlear groove angle analysis, all specimen stages were divided equally into three groups to achieve an equal CRL range (Group 1, 21–80 mm, n = 9; Group 2, 81–140 mm, n = 9; and Group 3, 141–200 mm, n = 6). This classification was employed to identify changes in trochlear groove angle and distal femoral morphology in Procrustes analysis associated with CRL growth. The Shapiro–Wilk test was used to confirm data normality. Subsequently, the Tukey–Kramer test was used to determine significant differences among groups. The relationship between patella angles A and B, flexion and extension joint motion, and femur length and CRL was reported using Spearman-rank correlation analysis. A smoothing spline was used to examine the trends of all angles and joint motions. In the Procrustes analysis, principal component analysis (PCA) and linear discriminant analysis (LDA) were performed on landmarks #1–3 related to the trochlear groove formation, as well as on the parameters that showed group differences among the CRL groups. Statistical analysis was conducted using JMP Pro version 16 (SAS Institute Inc., Cary, NC, USA), and statistical significance was set at *p* < 0.05.

## Results

### Morphogenesis of bones comprising the knee joint

We examined hematoxylin-eosin-stained tissue sections, CT, and MR images of CS18–CS22 knee joint samples to identify the ideal timing for image analysis. At CS18 and CS19, we identified femoral and tibial primordia. However, we could not identify the patella, while tissue boundaries were histologically indistinct on CT images. At CS20, we identified patellar primordia, with the boundary between the patella and femur being histologically clear. However, these remained unidentified on CT images. On CT images, we simultaneously identified the tibia, femur, and patella; nonetheless, the cartilage portion of the patella remained unclear at CS20–CS21. We observed that the borders between the patella and femur were sufficiently distinct to allow for image analysis at CS22 on 7T MR images (S2 Fig). Therefore, we conducted MR image analyses of the patella using CS22 samples. Femur length was strongly positively correlated with crown-rump length (CRL), indicating normal development of the specimens (S3A Fig).

**Fig 3:**
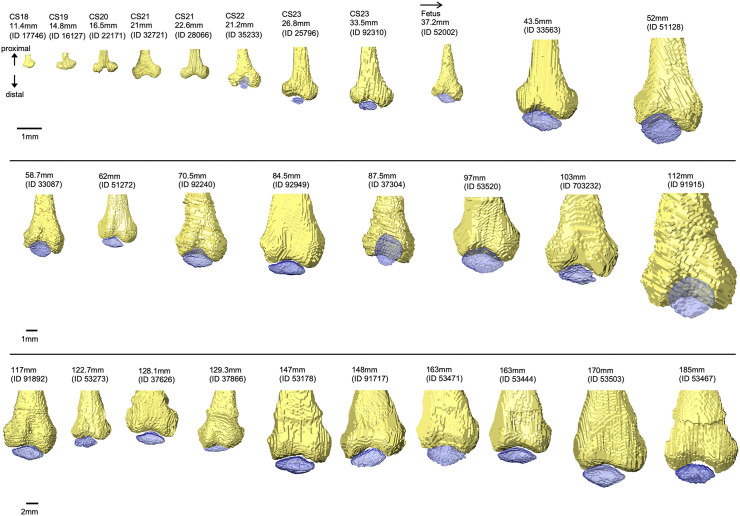
Development of distal femur bone and patella morphology during embryonic and fetal stages on the parallel plane of the femur axis. The images show the femur and patella (blue). The surfaces of the underlying subchondral bone of the femoral condyle and trochlear groove were observed. All images were obtained from the knee joint of the right lower limb.

We first observed the cartilaginous femur in CS18 specimens using CT images. We detected the contour with high signal intensity. In contrast, we observed the internal portion with slightly lower signal intensity; however, the line of the contours remained indistinct. The 3D reconstruction of the femur indicated a “T-shape,” as the femur intercondylar fossa and trochlear groove were not specified at CS18–CS19, with their formation beginning at CS20. The intercondylar fossa at CS22 resembled that in adults. The trochlear groove continued to form until the fetal CRL reached approximately 120 mm, after which the groove was still observed (Fig 3).

**Fig 4:**
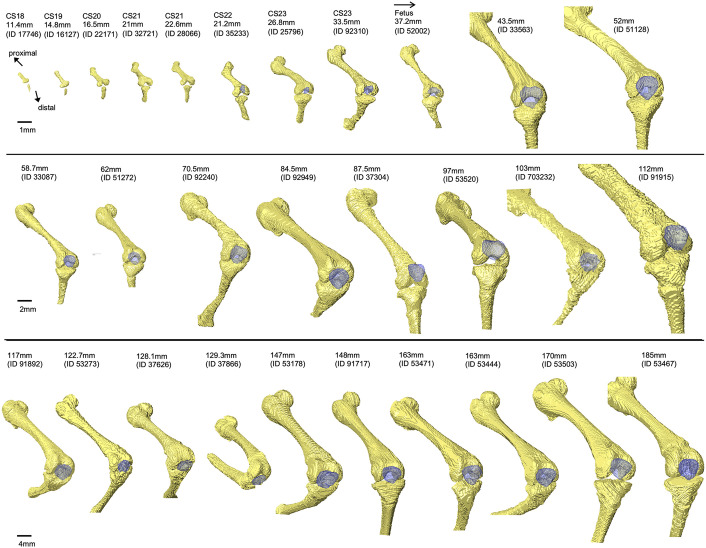
Overview of knee joint development during embryonic and fetal stages. The images show the femur, patella (blue), and tibia. Bone was reconstructed from all specimens, while the patella was reconstructed from Carnegie stage 22 specimens. All images were of the knee joint of the right lower limb, omitting the fibula.

Similar to the femur, we observed cartilaginous tibia at CS18. As the bone surface borders were indistinct, we could not observe the medial and lateral condyles of the proximal tibia in the 3D reconstruction of the tibia. At CS21, the formation of the medial and lateral condyles began, and the changes in the proximal tibia shape continued, with the 43.5 mm CRL fetus specimen resembling that of the adult. We also observed the formation of a rough tibial surface at 43.5 mm CRL (Fig 4).

Patella formation continued after the embryonic period. We observed the articular surface of the patella with the trochlear groove of the femur in a CS23 embryo at 26.8 mm CRL (Fig 4).

### Trochlear groove angle of the joint between the femur and patella

**Fig 5:**
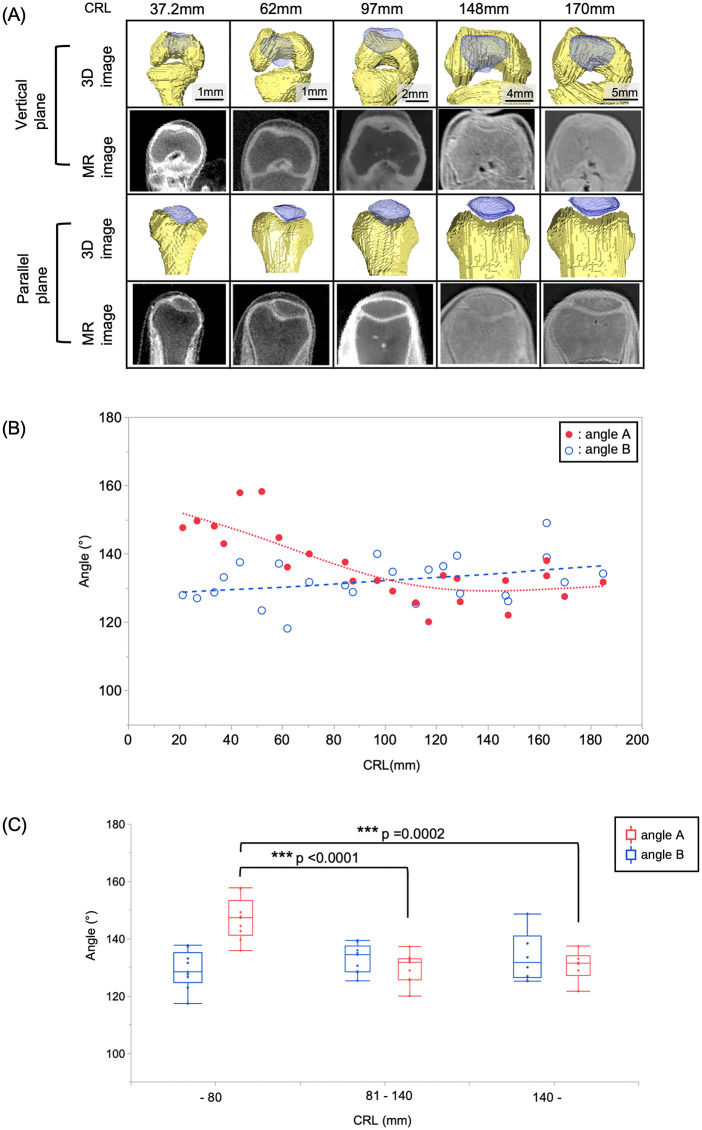
Trochlear groove measurements. **(A)** 3D reconstructed images of Amira and magnetic resonance (MR) scans on parallel and vertical planes at five stages of crown-rump length (CRL). The upper two lanes show the vertical plane, while the lower two lanes show the parallel plane. **(B)** Trochlear groove angle A (red dots) decreased from approximately 160° to 130° during 21.2–100 mm crown-rump length (CRL) and remained constant at 185 mm CRL. In contrast, angle B (blue dots) remained constant at approximately 130° during 21.2–185 mm CRL. Based on the distribution of the data, polynomial fitting used for Angle A and linear fitting were used for Angle **B. (C)** Chronological changes in the trochlear groove. Angle A exhibited significantly larger values in the early than later CRL stages. Angle B showed no significant differences among the groups.

We found that angle A decreased from approximately 160° to 130° during 26.8–120 mm CRL and remained constant at 185 mm CRL (Figs 4, 5A-C). In contrast, we noted that angle B remained constant at approximately 130° during 26.8–185 mm CRL (Figs 3, 5A-C). Analysis of the three groups by stage showed that only angle A exhibited significantly larger angles in the early than later CRL phases (Fig 5C, p < 0.05). However, we did not detect any significant differences in angle B across all groups. These angle raw data can access via S1 Table.

### Procrustes analysis

**Fig 6:**
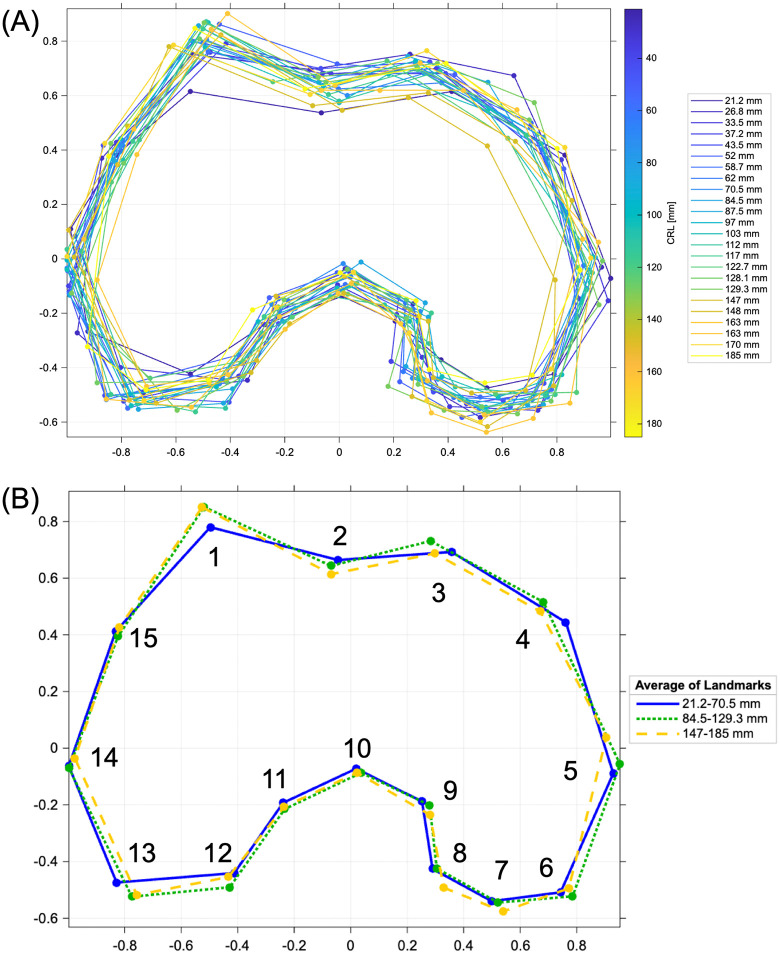
Reconstructed Procrustes shape coordinates for the distal femoral epiphysis. **(A)** Distal femoral shape was reconstructed from all specimens and adjusted to size for morphological comparison. **(B)** The reconstructed Procrustes image shows the formation of the trochlear groove (landmark #2) and sharpness of the peak of the lateral femoral condyle (landmark #1) with increasing crown-rump length (CRL). The numbers next to each dot indicate the landmark number.

We performed Procrustes analysis to assess femoral morphology independently of bone size and rotation. We detected differences in the morphology of the distal femur across its developmental stages (Fig 6A). The morphological changes associated with increased CRL can be seen in S1 File. In a three-group comparison analysis based on CRL size at each landmark angle, it showed that lateral trochlear peak angle (landmark #1) and trochlear groove angle (landmark #2) became significantly sharped in Group 2 and 3 (after 81 mm CRL) compare with the Group 1 (21–80 mm CRL). In addition, medial point of the anterior part of the lateral condyle (landmark #15) became significantly rounded in Group 2 compare with Group 1, and remained rounder in Group 3 (Fig 6B, Table 3 and S2 Table). The angle data and coordinate of all landmarks can access via S2 and S3 Table, respectively.

**Table 3:** Results of the three-group comparison of landmark angles.

Landmarks No.	CRL group		
	1	2	3
1	118.47 ± 2.15†‡	99.08 ± 2.15†	97.04 ± 2.63‡
2	161.40 ± 2.66†‡	140.47 ± 2.66†	142.09 ± 3.26‡
3	144.67 ± 3.90	137.41 ± 3.90	143.12 ± 4.78
4	138.06 ± 2.55	142.89 ± 2.55	143.67 ± 3.12
5	138.52 ± 2.90	135.22 ± 2.90	138.81 ± 3.55
6	122.88 ± 3.76	114.88 ± 3.76	121.46 ± 4.61
7	141.94 ± 3.82	144.71 ± 3.82	140.94 ± 4.67
8	129.65 ± 8.76	127.85 ± 8.76	122.70 ± 10.73
9	233.49 ± 5.74	237.65 ± 5.74	228.81 ± 7.03
10	231.14 ± 3.29	229.72 ± 3.29	235.53 ± 4.03
11	210.53 ± 3.90	209.33 ± 3.90	207.09 ± 4.78
12	128.81 ± 3.66	131.32 ± 3.66	137.86 ± 4.48
13	108.88 ± 4.00	110.98 ± 4.00	106.88 ± 4.89
14	136.67 ± 2.86	132.30 ± 2.86	132.49 ± 3.50
15	157.69 ± 2.64*	167.15 ± 2.64*	165.70 ± 3.23

†: Group1 vs 2, p < 0.01, ‡: Group1 vs 3, p < 0.01; *Group1 vs 2, p < 0.05.

Only 2 is an exterior angle, the others are interior angles.

As a result of performing PCA and LDA analysis on the angles of landmarks #1, 2, 3, and 15 for the three CRL size groups mentioned above, it revealed that the first two principal components accounted for over 82% of the total variance (All samples, PC1: 62.1%, PC2: 22.6%; Group1, PC1: 62.8%, PC2: 19.8%; Group 2, PC1: 71.3%, PC2: 15.6%; Group 3, PC1: 63.1%, PC2: 19.0%). For PC1, landmark #3 showed the highest positive loading in Groups 1 and 2, while landmark #2 showed the highest positive loading in Group 3. Landmark #15 exhibited negative loadings in Groups 1 and 2 but shifted to a positive loading in Group 3. In contrast, landmark #1 demonstrated the opposite trend. In the analysis of all samples, landmark #2 showed the highest positive loading. The LDA results indicated a different trend between Group 1 and Groups 2 and 3 (S2 File).

### Morphological comparison of the hamstring and quadriceps muscles

To examine the relationship between the skeletal development of the knee joint and maturation of the surrounding muscles, we made 3D reconstructions of the muscles in the fetal period, a period posterior to CS23 [22], when the main muscles resemble those of adults. However, in two individuals (129 mm and 180 mm CRL), we could not achieve the 3D reconstruction of muscles because the muscle boundaries were unclear due to low resolution. Hence, we did not include these samples in this analysis.

**Fig 7:**
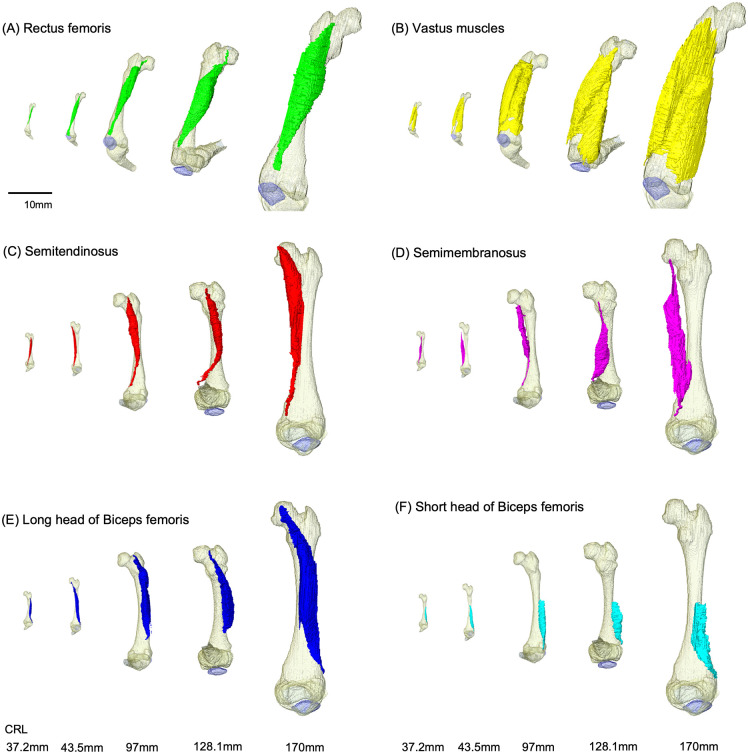
3D reconstructed models of the muscles involved in knee joint movement with leg skeleton during 37.2–170 mm crown-rump length (CRL). Samples at 37.2, 43.5, 97, 128.1, and 170 mm CRL are shown. (A) Rectus femoris, (B) vastus, (C) semitendinosus, (D) semimembranosus, (E) long head of biceps femoris, and (F) short head of biceps femoris muscle.

**Fig 8:**
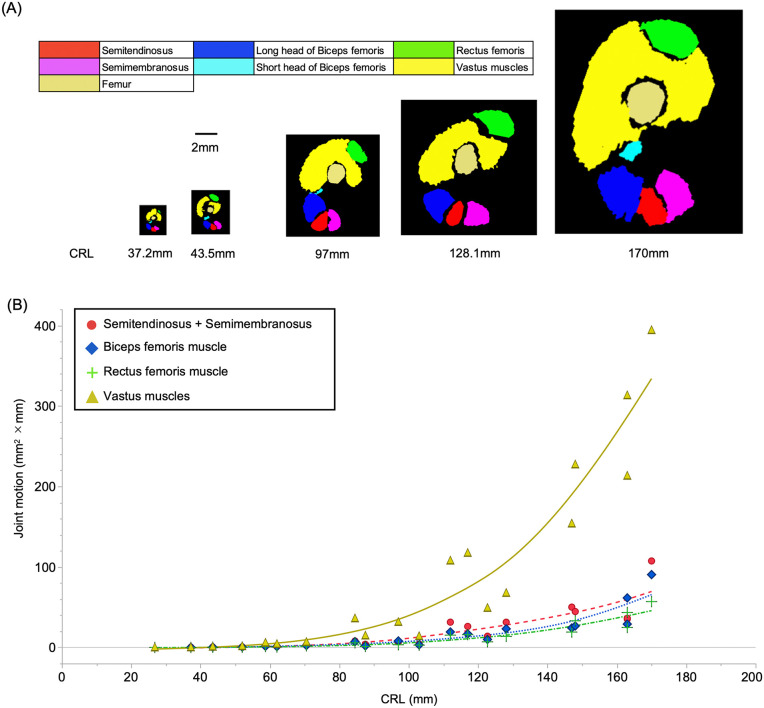
Chronological changes in the cross-section area (CSA) at the center of the thigh (A) and joint motions by each thigh muscles (B). The CSA of all muscles increased gradually. The CSA of the vastus muscle increased dramatically from 100 mm crown-rump length (CRL). All joint motions increased slowly during 30–100 mm CRL, whereas rapidly after approximately 100 mm CRL. The vastus muscle activity increased dramatically. Based on the distribution of the data, exponential approximation was applied for each muscle’s joint motion data.

For all muscles, the linearly increasing trend of the line of action (LA) in proportion to CRL was similar (S3B Fig); nevertheless, muscle volume and length increased differentially among muscles (Figs 7, S2C). The cross-sectional area (CSA) was also increased in proportion to the CRL in a quadratic curve (Figs 8A, S3D).

For the hamstring muscle, LA increased in proportion to CRL (S3B Fig). Muscle volume increased very slowly up to a specimen with a 70.5 mm CRL, gradually increasing as it grew (Fig 7). The increase in muscle volume of the short head of the biceps femoris muscle was very small compared with that of the semitendinosus and semimembranosus muscles and the long head of the biceps femoris muscle. Raw data for the muscles analysis of each sample are available via S1 Table.

In addition, the CSA of the quadriceps rectus femoris muscle increased slightly, whereas that of the vastus muscles increased rapidly from 60 to 175 mm CRL (Figs 8A, S2D, and S1 Table). The 3D reconstruction images also showed that the volume of the vastus femoris muscle increased significantly more compared with that of other muscles (Figs 7, 8A).

### Muscle force and correlation between trochlear groove angle and joint motions

For knee flexor muscles, the hamstring hypothetical joint motion increased slowly during 30–100 mm CRL, whereas rapidly after approximately 100 mm CRL. For knee extensor muscles, the more markedly increased in the vastus muscles hypothetical joint motion after 100 mm; however, the force of the rectus femoris was slightly increased (Fig 8B). When comparing the two muscle categories in hamstring, the joint motion of the semitendinosus and semimembranosus muscles often slightly exceeded that of the biceps femoris muscle (Fig 8B). We detected a significant negative correlation between angle A and all joint motions (Table 4). The correlation coefficients were fairly close, but slightly greater in the knee extensor group than in the flexor group (extensor group: *ρ* = −0.69, *p* < 0.001; flexor group: *ρ* = −0.66, *p* = 0.002). A scatter plot of trochlea Angle A or B and muscle motions are shown in S3 File.

**Table 4:** Correlation between patella angles A, B, and flexion and extension joint motion.

Variables	vs. Variables	ρ	p-value
Angle A	Extensor muscles	−0.69	<0.001*
	Flexor muscles	−0.66	0.002*
	Rectus femoris muscle	−0.67	0.001*
	Vastus muscles	−0.69	<0.001*
	Semitendinosus and Semimembranosus	−0.7	<0.001*
	Biceps femoris	−0.65	0.002*
Angle B	Extensor muscles	0.2	0.39
	Flexor muscles	0.2	0.398

*: p < 0.05.

No significant correlations were detected between angle B and each muscle.

## Discussion

This study examined the developmental process of the musculoskeletal structure around the knee joint during the embryonic and early fetal stages from kinematic and morphological perspectives. The trochlear groove angle stabilized at approximately 120 mm crown-rump length (CRL). All joint motions in the thigh, especially the vastus muscles, increased slowly during 30–100 mm CRL, whereas rapidly after approximately 100 mm CRL. Although there is no direct evidence between muscle development and trochlear groove formation, and the possibility of a spurious correlation cannot be excluded, a negative correlation was identified between hypothetical joint motion and trochlear groove angle A. This study focused on the relationship between morphological changes in knee joint components and joint motion including muscle development from the human embryonic to fetal period.

The morphological changes in bones and muscles during embryonic growth matched those reported in previous studies [20,22].

Regarding trochlear groove formation, our results showed that the trochlear groove angle remained constant from approximately 120 mm CRL (approximately gestational age (GA) 130 d). Procrustes analysis revealed that the protrusion of the lateral femoral condyle became more pronounced with growth and that the angular position that contributes to femoral morphology differs depending on the stage of growth. Compared with previous studies using adult specimens under conditions similar to ours, that is, measurements were conducted on the plane +15° anterior to the perpendicular to the femoral axis, the trochlear groove angle values and higher height of the lateral than medial trochlear matched adult morphology [34]. Studies have shown that during childhood (ages 3–16 years), the trochlear groove angle remains constant, with the height of the lateral trochlear groove exceeding that of the medial and continuing to grow across all ages studied [17]. Similar results were obtained in a previous study on the embryonic period despite lacking a time series [27]. Our study revealed the angular stabilization of the trochlear groove over time from embryonic to fetal stages and that prenatal protrusion of the lateral femoral condyle occurs before birth.

Our findings indicate that trochlear groove morphogenesis proceeds through region-specific mechanisms.

Regarding the timing of trochlear groove stabilization and the timing of the increase in muscle mass and joint motion, our findings showed that stabilization of the patellar trochlear angle occurred around a 100–120 mm CRL, after which muscle mass and joint motion rapidly increased. In addition, a negative correlation was observed between joint motion and trochlear groove angle A.

This interpretation is consistent with previous research, which reported that patellofemoral parameters such as patellar height and trochlear morphology in newborns are strongly influenced by intrauterine position and mechanical factors, suggesting that patellofemoral morphology remains responsive to mechanical forces throughout development [40]. Furthermore, previous animal study using muscle dysgenesis model have shown that the patella primordium forms independently of joint motion, but subsequent muscle contraction and mechanical stress promote chondrocyte differentiation at the patellofemoral boundary [3]. Besides, although the joints are different, reduced gluteal muscle volume compare with health side has been reported in individuals with hip dysplasia [41,42], suggesting that normal muscle development follows proper morphological formation. Considering the similarity of the timing of the muscle development and groove angle stabilization, it is possible that increased fetal movement after the completion of the embryonic patellar primordium formation promoted the stabilization of patellofemoral joint morphogenesis, resulting in the formation of a biomorphic shape similar to the adult trochlear groove [43]. It is suggested that the subsequent stabilization of joint morphology enables smooth joint motion, which leads to an increase in muscle mass and joint motion.

In contrast, Angle B, measured on the plane parallel to the femoral axis, did not show a significant correlation with joint motion, suggesting that the trochlear groove angle is established earlier and is less affected by dynamic patellofemoral movement.

Although the trochlear surface on this plane corresponds to patellofemoral joint surface, it also forms part of the femorotibial contact surface in extension. Therefore, it is possible that the articular interface with the tibia develops earlier than that with the patella, leading to the early stabilization of Angle B observed in this study.

This interpretation aligns with the developmental framework described in previous research, which reported that femorotibial joint surfaces begin to differentiate early in the fetal period, preceding the maturation of patellofemoral structures [44].

Regarding the development of muscles involved in knee joint movement, our results showed that muscle mass and length increased in all muscles analyzed at approximately 60–130 mm CRL. The vastus muscles showed a significant increase in muscle mass, far exceeding that of other thigh muscles (rectus femoris and hamstrings). This finding aligned with that of previous studies [22]. Muscle mass correlates with joint motion. Previous research has shown that age-related muscle loss depends on muscle quality: changes in muscle fiber type and perimuscular tissue such as adipose and fibro tissue [45]. However, as we conducted this study on fetuses and embryos, muscle strength may be influenced by muscle mass rather than muscle quality. Considering that muscle mass and the hypothetical joint motion are greatest in the vastus femoris and exceed the total hamstring strength at any point, this information suggested that extension may exceed flexion in the knee joint motion that may have developed from 60 mm CRL onward, and that this motion may influence bone morphology.

Although the relationship between GA and CRL is controversial, during the first trimester, especially between 9 and 13 weeks GA, linear growth evaluated by CRL is rapid, and thus GA can be estimated accurately by CRL. Whereas, in later pregnancy, CRL variation is greater, resulting in less accurate estimation of GA [46]. A large international study on the relationship between GA and CRL reported the following: 11weeks GA includes 49.39 ± 6.62 mm CRL, 12 weeks GA includes 60.78 ± 7.07 mm CRL, and 13 weeks GA includes 72.53 ± 7.29 mm CRL [47]. In other words, the specimens analyzed in this study include not only those from the embryonic stage and 9–13 weeks GA, but also, based on the sample information, those up to 19 weeks GA. During normal development, the nervous and musculoskeletal systems develop in coordination throughout the fetal life, enabling progressively complex limb movements. The differentiation of specific movement patterns within the 8–14-week GA period results from simple limb movements at 9–10 weeks GA and complex limb movements at 11–12 weeks GA, which derive from the upper spinal cord [48,49]. Studies have also shown that the number of axonal synapses of motor neurons increases rapidly at 14–15 weeks GA [50]. When applied to the present study, joint motion began to gradually increase around 60 mm CRL, corresponding to approximately 12 weeks, and a rapid increase in both muscle mass and joint motion was observed during 87.5–120 mm CRL, around 14–15-weeks GA, which is reported as the period of rapid motor neuron proliferation. Among them, the hypothetical joint motion of the quadriceps is increased in the range of 60–175 mm CRL, which corresponds to after 12 weeks GA and coincides with the period of rapid development of motor neurons, when limb movements begin. Thus, the temporal changes in joint motion and muscle development observed in this study are considered to be consistent with the development of the nervous system which reported in previous study.

Fetal movement and joint motion play an essential role in normal skeletal development, including patella formation [2–4,9,51]. At the molecular level, previous animal studies have shown that while muscle contraction is not required for the initiation of patella formation, it is essential for maintaining the fate of joint progenitor cells [3,51]. The patella arises from a distinct population of Sox9-positive and Scx-positive progenitor cells, whose differentiation depends on TGFβ and BMP4 signaling [3]. Mechanical stimulation has been demonstrated to regulate the expression of Sox9, Scx, and TGFβ-related genes during joint morphogenesis [7], and to promote chondrocyte differentiation via integrin-mediated activation of the TGFβ pathway [8]. Furthermore, mechanosensitive genes such as BMP2 and CD44, as well as patterning-related genes including WNT9a and BMP4, contribute to the signaling processes that coordinate developing joint morphology [5,6]. Although molecular analysis was not performed in the present study, our findings suggest that these mechanotransduction pathways could also underlie the morphological development of the trochlear groove. Future studies are expected to examine the relationship with these pathways.

This study had certain limitations. First, we did not assess the sartorius muscle as part of the knee flexion muscles. To capture the lower limb movements controlled by the thigh muscles in more detail, future analyses should include the sartorius muscles. Second, since fixed specimens are used, the actual movements of the fetus are unknown, and the causal relationship between joint movement and bone morphogenesis remains hypothetical. However, there have been few studies conducted with human specimens from this perspective, and it is expected that the current results will provide new insights. Third, although we used MR imaging to confirm detailed muscle morphology and attachments, the joint motions calculated in this study are hypothetical and do not necessarily reflect actual movements. Finally, in the fetal period, large individual differences exist in body size, such as CRL and femur length relative to GA. Therefore, correlating joint motion and GA in more detail requires a large amount of sample data. In the future, current technologically advanced 3D ultrasound may enable more accurate measurements in vivo [9].

## Conclusions

Our study revealed that the trochlear groove angle stabilized at approximately 120 mm CRL, coinciding with the onset of fetal movement and increasing muscle mass and force. The correlation between angle change, increased muscle strength, and fetal movement timing, particularly in the direction of extension, suggested that joint movement may contribute to trochlear groove formation.

## Supporting information

S1 Fig Scheme of Hypothetical joint motion of biceps femoris. Lateral view of the right lower limb in hip and knee flexion position. Hypothetical joint motion is calculated by multiplying CSA and LA. CSA: Muscle cross sectional area, LA: line of action, Pel: Pelvic, Fem: Femur, Tib: Tibia, Blue muscle: biceps femoris, red muscle: semitendinosus.
(TIFF)

S2 Fig Comparison of histological, computed tomography (CT), and magnetic resonance (MR) image observations in Carnegie stages (CS) 20–22. Histological (A–C), CT (D and E), and MR (F) images showing that the initial patella formation and boundaries between the patella and femur were only histologically distinct by CS21. Sufficiently distinct patella borders for image analysis are observed at CS22 on MR images (yellow circle). F: femur bone; T: tibia bone; P: patella; p: cartilage primordium of the patella.
(TIFF)

S3 Fig Relationship between crown-rump length (CRL) and other parameters. The relationship between (A) CRL and femur length, (B) CRL and the line of action (LA) of each muscle, (C) CRL and muscle length of each muscle, and (D) CRL and average cross-sectional area (CSA). Femur length was strongly positively correlated with CRL. The hamstring muscle parameters increased with increasing CRL; the volume increased very slowly up to 70.5 mm CRL, after which it increased rapidly. The increase in muscle volume of the short head of the biceps femoris muscle was very small compared with that of the semitendinosus and semimembranosus muscles and the long head of the biceps femoris muscle. Regarding the quadriceps muscle, the CSA of the quadriceps rectus femoris muscle increased very slightly, whereas that of the vastus muscles increased rapidly during 60–140 mm CRL.
(TIFF)

S1 Table Raw data of trochlear groove angle and muscle analysis. (PDF)

S2 Table Raw angle data for each landmark in Procrustes analysis. (PDF)

S3 Table Coordinates for 15 landmarks in Procrustes analysis. (PDF)

S1 File Image of comparison of distal femoral epiphysis shapes without size adjustment. (PDF)

S2 File The results of principal component analysis (PCA) and linear discriminant analysis (LDA) of Procrustes analysis. This file contains the results for the whole sample and each CRL group: score plots and loading tables from the PCA analysis, and canonical plots from the LDA analysis.
(PDF)

S3 File A scatter plot of trochlea Angle A or B and muscle motions. (PDF)
