# Supplementary figures and images for: Morphogenetic development of trochlear groove and thigh muscles from embryo to fetus in humans
Source: PLoS One. 2026 Feb 2;21(2):e0339167. doi: 10.1371/journal.pone.0339167 (PMC12863510; doi:10.1371/journal.pone.0339167)

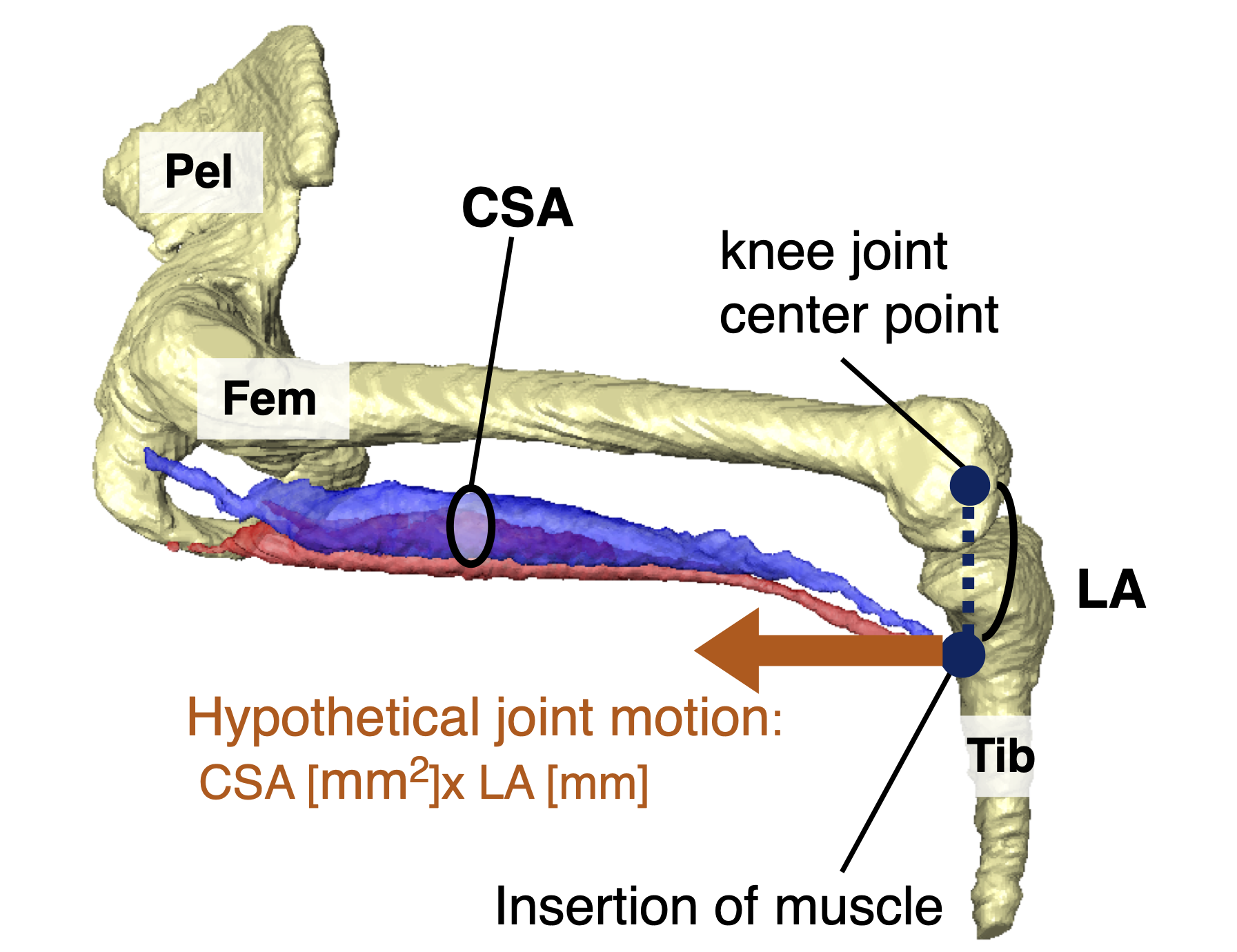

Supplement: S1 Fig — Lateral view of the right lower limb in hip and knee flexion position. Hypothetical joint motion is calculated by multiplying CSA and LA. CSA: Muscle cross sectional area, LA: line of action, Pel: Pelvic, Fem: Femur, Tib: Tibia, Blue muscle: biceps femoris, red muscle: semitendinosus. (TIFF) [file pone.0339167.s001.tiff]

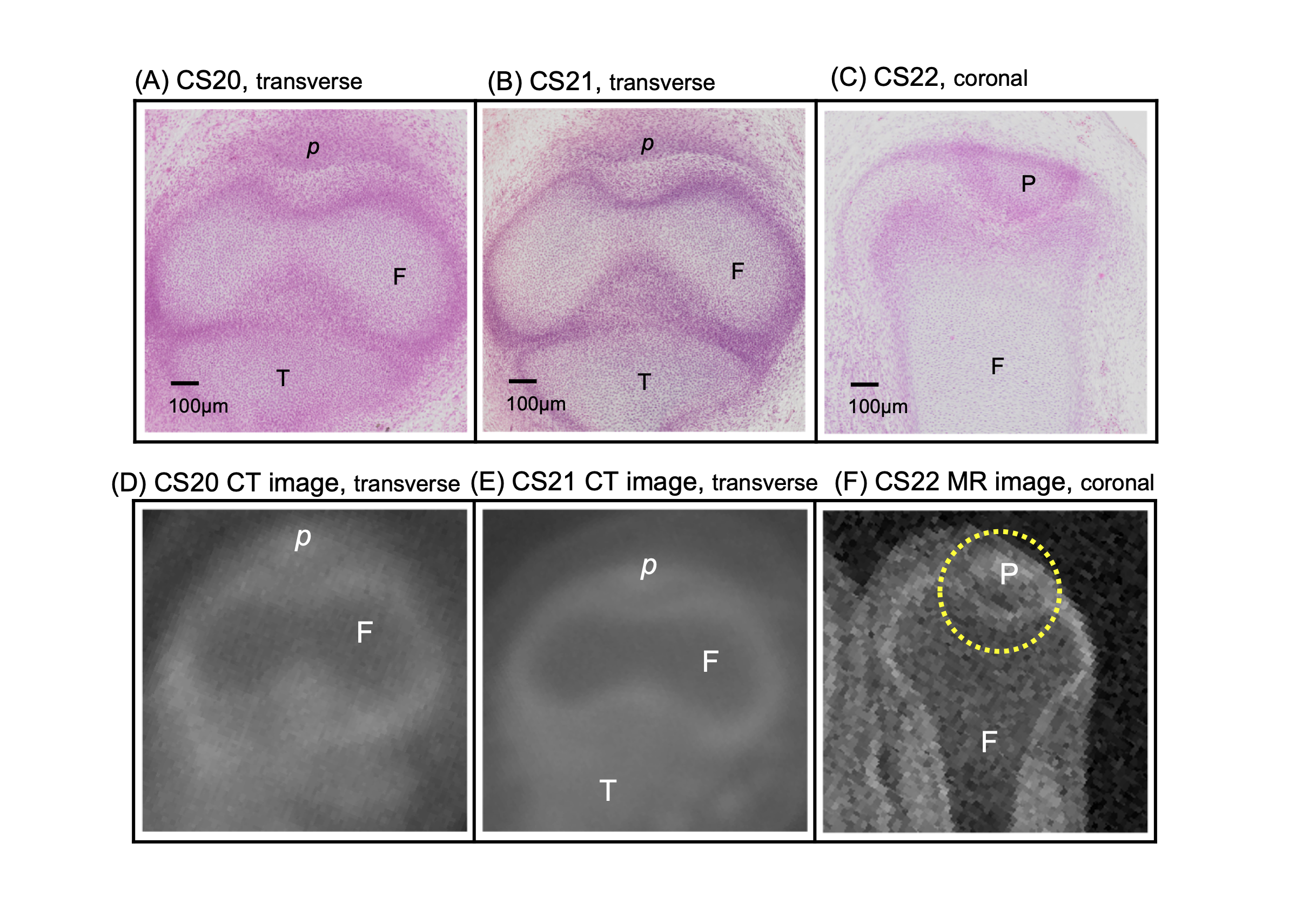

Supplement: S2 Fig — Histological (A–C), CT (D and E), and MR (F) images showing that the initial patella formation and boundaries between the patella and femur were only histologically distinct by CS21. Sufficiently distinct patella borders for image analysis are observed at CS22 on MR images (yellow circle). F: femur bone; T: tibia bone; P: patella; p: cartilage primordium of the patella. (TIFF) [file pone.0339167.s002.tiff]

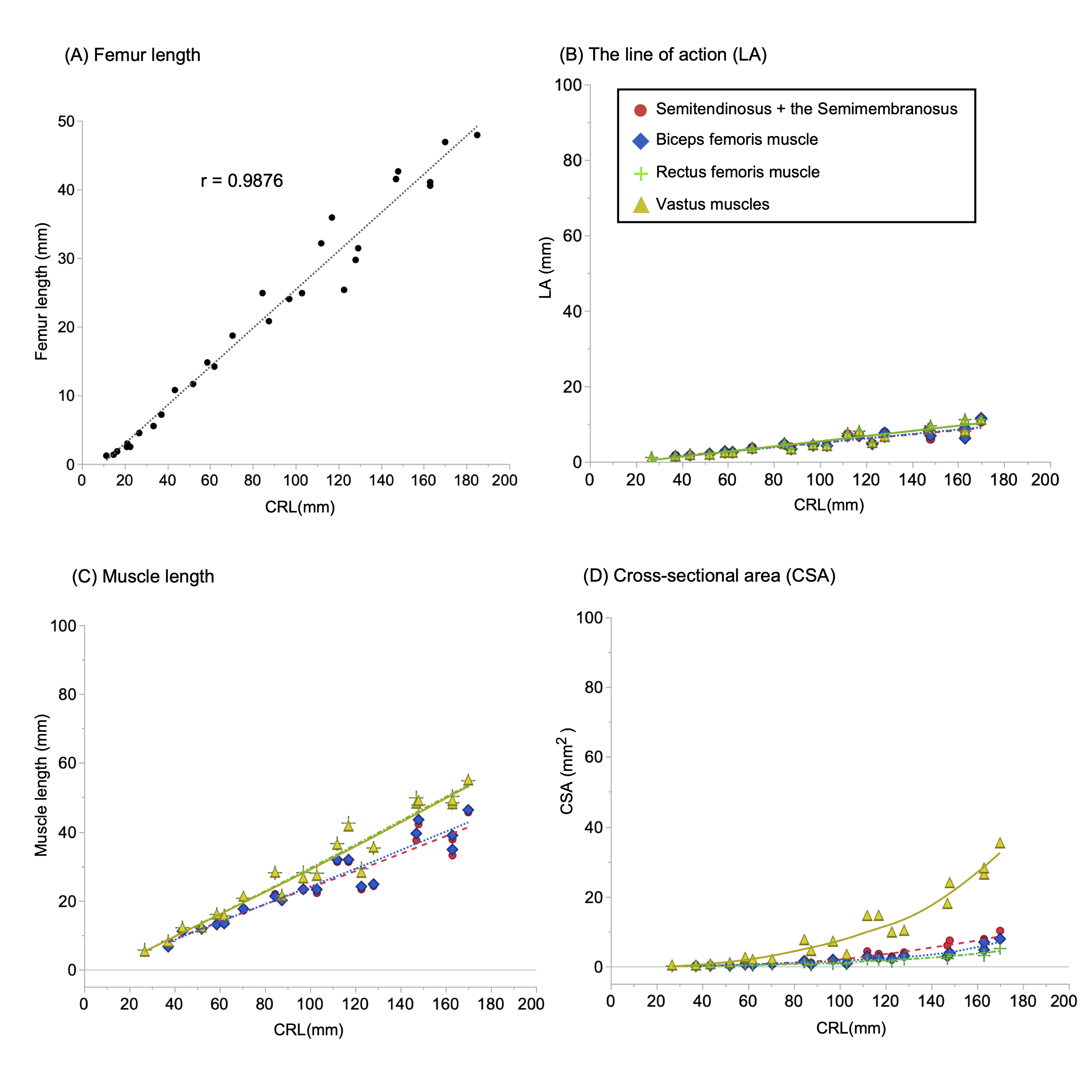

Supplement: S3 Fig — The relationship between (A) CRL and femur length, (B) CRL and the line of action (LA) of each muscle, (C) CRL and muscle length of each muscle, and (D) CRL and average cross-sectional area (CSA). Femur length was strongly positively correlated with CRL. The hamstring muscle parameters increased with increasing CRL; the volume increased very slowly up to 70.5 mm CRL, after which it increased rapidly. The increase in muscle volume of the short head of the biceps femoris muscle was very small compared with that of the semitendinosus and semimembranosus muscles and the long head of the biceps femoris muscle. Regarding the quadriceps muscle, the CSA of the quadriceps rectus femoris muscle increased very slightly, whereas that of the vastus muscles increased rapidly during 60–140 mm CRL. (TIFF) [file pone.0339167.s003.tiff]

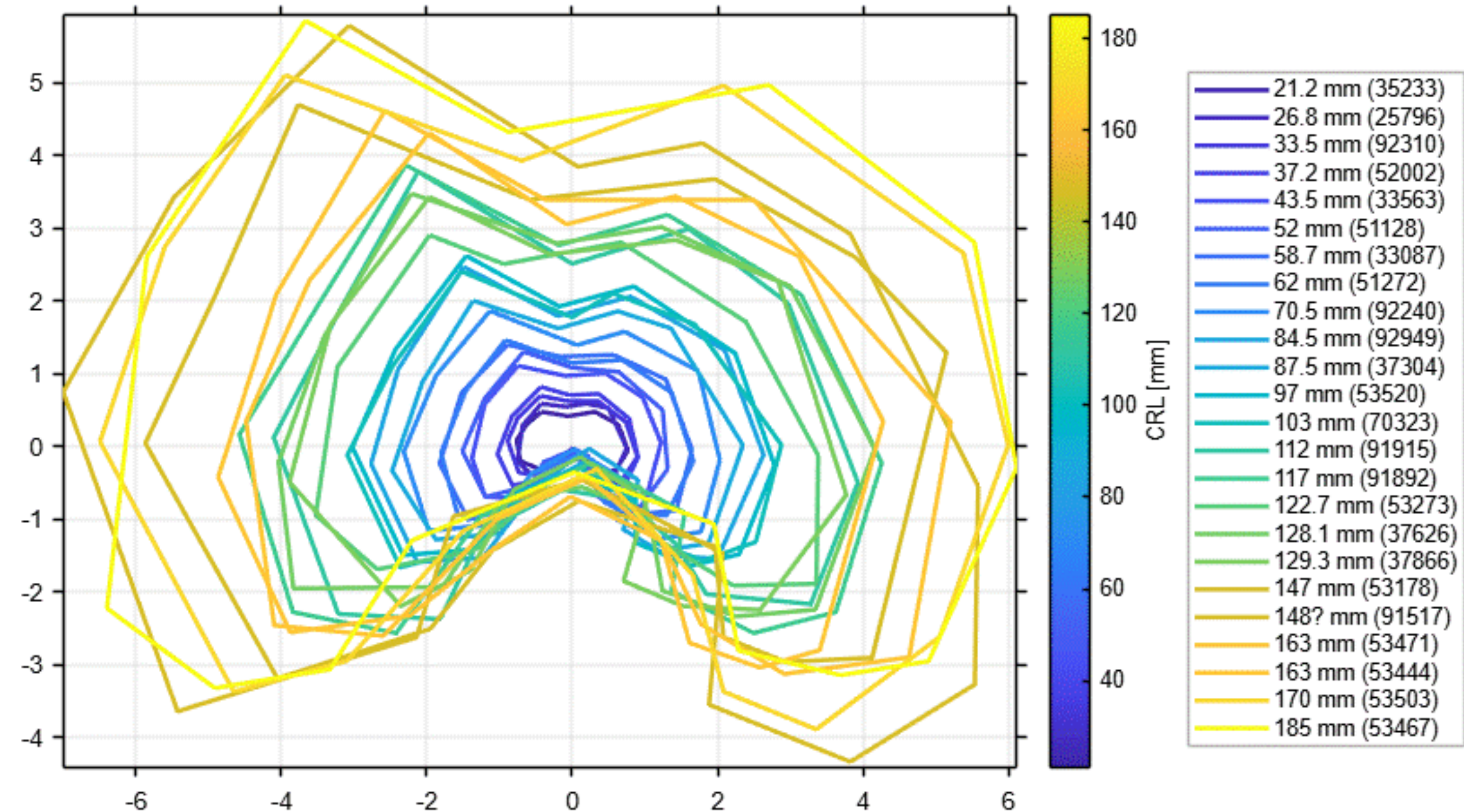

Supplement: S1 File — (PDF) [file pone.0339167.s007.pdf]
