# Supplementary material for: Morphogenetic development of trochlear groove and thigh muscles from embryo to fetus in humans
Source: PLoS One. 2026 Feb 2;21(2):e0339167. doi: 10.1371/journal.pone.0339167 (PMC12863510; doi:10.1371/journal.pone.0339167)
Supplement: S2 Table — (PDF) [file pone.0339167.s005.pdf]

Specimen ID	GA (day)	CRL (mm)	**1**	**2**	**3**	**4**	**5**	**6**	**7**	**8**	**9**	**10**	**11**	**12**	**13**	**14**	**15**
17746	52	11.4	-	-	-	-	-	-	-	-	-	-	-	-	-	-	-
16127	59	14.8	-	-	-	-	-	-	-	-	-	-	-	-	-	-	-
22171	65	16.5	-	-	-	-	-	-	-	-	-	-	-	-	-	-	-
32721	65	21.0	-	-	-	-	-	-	-	-	-	-	-	-	-	-	-
28066	80	22.6	-	-	-	-	-	-	-	-	-	-	-	-	-	-	-
35233	56	21.2	130.19	198.42	141.43	139.73	128.97	132.54	136.5	171.37	195.22	225	193.71	123.83	122.26	138.46	162.36
25796	84	26.8	123.55	196.15	159.62	126.15	131.76	123.9	139.68	133.21	229.33	240.67	211.91	121.73	131.08	124.22	147.04
92310	71	33.5	125.3	190.92	143.29	145.15	128.83	120.19	121.79	181.33	212.86	234.32	205.37	120.29	115.53	137.42	157.41
52002	104	37.2	118.38	198.67	137.98	142.36	141.58	123.77	142.03	112.96	250.02	228.01	221.25	129.56	103.3	133.19	156.92
33563	117	43.5	118.94	195.62	143.64	144.72	143.18	127.51	150.43	108.51	232.47	234.12	209.63	119.09	112.13	147.91	152.11
51128	82	52.0	111.28	193.24	144.21	135.87	139.16	138.49	137.73	115.56	246.43	227.98	221.8	132.86	101.69	130.43	163.29
33087	90	58.7	121.03	194.82	152.68	135.57	145.34	110.44	149.55	119.8	244.79	221.66	211.81	141.18	102.14	135.75	153.43
51272	99	62.0	109.16	202.31	143.21	141.11	136.17	114.68	153.99	115.79	245.97	226.87	212.27	141.61	90.06	143.25	163.54
92240	93	70.5	108.41	217.2	135.98	131.84	151.66	114.37	145.8	108.33	244.3	241.65	207.05	129.14	101.72	139.4	163.15
92949	63	84.5	94.88	222.5	135.4	140.12	135.73	108.99	153.65	104.39	254.4	247.68	197.73	130.96	111.26	138.9	163.42
37304	101	87.5	103.76	214.29	145.87	139.54	129.1	117.21	136.84	134.47	235.56	232.47	206.49	132.16	116.57	128.66	167.02
53520	102	97.0	98.4	223.72	131.49	142.75	138.99	124.94	143.93	112.83	242.65	228.64	216.24	127.17	110.43	131.07	166.75
70323	N.D.	103.0	103.47	232.81	129.19	146.25	129.57	124.28	140.14	140.97	214.78	231.13	203.45	144.61	100.54	127.84	170.97
91915	109	112.0	88.58	227.42	126.54	148.13	137.55	105.76	126.4	198.44	211.52	217.07	216.12	113.12	112.9	132.45	178.03
91892	136	117.0	94.3	223.38	133.36	146.48	137.38	121.45	138.55	107.42	251.73	233.72	208.43	114.29	118.27	131.92	179.32
53273	120	122.7	103.62	211.78	137.01	150.92	150.8	92.06	147.22	136.06	237.65	225	194.58	148.93	127.88	123.1	153.38
37626	136	128.1	102.44	207.82	146.35	142.1	132.45	115.04	155.96	119.22	237.37	212.85	224.94	125.39	96.16	153.33	168.59
37866	131	129.3	102.23	212.05	151.5	129.69	125.42	124.2	159.67	96.87	253.15	238.91	215.98	145.23	104.79	123.41	156.89
53178	123	147.0	91.17	208.67	144.46	162.92	122.6	106.4	155.47	117	246.37	228.18	201.96	146.26	101.34	125.41	181.78
91517	136	148.0	103.85	222.41	137.75	148.4	152.16	122.84	126.1	106.96	247.43	223.79	236.86	124.21	92.48	130.67	164.08
53471	138	163.0	95.3	228.16	139.62	142.96	137.47	121.87	144.32	124.71	213.98	245.38	201.11	128.59	103.38	147.92	165.24
53444	133	163.0	91.69	208.89	180	131.59	128.4	107.6	141.44	145.98	219.11	240.04	184.49	149.28	106.96	137.45	167.07
53503	N.D.	170.0	105.08	220.37	124.56	138.06	147.28	146.52	120.96	126.99	207.26	252.69	203.4	147.08	102.86	133.45	163.44
53467	N.D.	185.0	95.11	218.99	132.34	138.07	144.94	123.53	157.34	114.57	238.7	223.1	214.72	131.76	134.24	120.02	152.58

All data is for the interior angles of landmarks.
