# Supplementary material for: Morphogenetic development of trochlear groove and thigh muscles from embryo to fetus in humans
Source: PLoS One. 2026 Feb 2;21(2):e0339167. doi: 10.1371/journal.pone.0339167 (PMC12863510; doi:10.1371/journal.pone.0339167)
Supplement: S3 Table — (PDF) [file pone.0339167.s006.pdf]

		Landmarks No.	1		2		3		4		5		6		7		8		9		10		11		12		13		14		15	
Specimen ID	GA (day)	CRL (mm)	X	Y	X	Y	X	Y	X	Y	X	Y	X	Y	X	Y	X	Y	X	Y	X	Y	X	Y	X	Y	X	Y	X	Y	X	Y
17746	52	11.4	-	-	-	-	-	-	-	-	-	-	-	-	-	-	-	-	-	-	-	-	-	-	-	-	-	-	-	-	-	-
16127	59	14.8	-	-	-	-	-	-	-	-	-	-	-	-	-	-	-	-	-	-	-	-	-	-	-	-	-	-	-	-	-	-
22171	65	16.5	-	-	-	-	-	-	-	-	-	-	-	-	-	-	-	-	-	-	-	-	-	-	-	-	-	-	-	-	-	-
32721	65	21.0	-	-	-	-	-	-	-	-	-	-	-	-	-	-	-	-	-	-	-	-	-	-	-	-	-	-	-	-	-	-
28066	80	22.6	-	-	-	-	-	-	-	-	-	-	-	-	-	-	-	-	-	-	-	-	-	-	-	-	-	-	-	-	-	-
35233	56	21.2	-0.42	-0.47	-0.05	-0.41	0.32	-0.47	0.64	-0.29	0.77	0.06	-0.21	0.19	-0.42	0.33	-0.71	0.21	-0.76	-0.08	-0.63	-0.29	-0.71	0.21	-0.76	-0.08	-0.63	-0.29	-0.76	-0.08	-0.63	-0.29
25796	84	26.8	-0.42	-0.59	-0.08	-0.54	0.20	-0.59	0.50	-0.52	0.75	0.02	-0.17	0.15	-0.26	0.35	-0.56	0.38	-0.75	0.21	-0.68	-0.29	-0.56	0.38	-0.75	0.21	-0.68	-0.29	-0.75	0.21	-0.68	-0.29
92310	71	33.5	-0.43	-0.69	-0.06	-0.62	0.36	-0.62	0.74	-0.33	0.90	0.14	-0.25	0.22	-0.39	0.40	-0.73	0.36	-0.88	-0.08	-0.75	-0.39	-0.73	0.36	-0.88	-0.08	-0.75	-0.39	-0.88	-0.08	-0.75	-0.39
52002	104	37.2	-0.43	-0.82	-0.03	-0.70	0.37	-0.72	0.78	-0.38	0.91	0.15	-0.23	0.15	-0.38	0.44	-0.82	0.54	-1.03	0.10	-0.83	-0.41	-0.82	0.54	-1.03	0.10	-0.83	-0.41	-1.03	0.10	-0.83	-0.41
33563	117	43.5	-0.74	-1.11	-0.09	-0.97	0.46	-1.00	0.97	-0.67	1.22	-0.05	-0.31	0.33	-0.56	0.73	-1.19	0.69	-1.37	0.17	-1.20	-0.58	-1.19	0.69	-1.37	0.17	-1.20	-0.58	-1.37	0.17	-1.20	-0.58
51128	82	52.0	-0.67	-1.30	-0.01	-1.08	0.64	-1.02	1.25	-0.50	1.31	0.31	-0.39	0.22	-0.56	0.53	-1.19	0.70	-1.51	0.07	-1.20	-0.66	-0.60	0.42	-0.99	0.88	-1.42	0.35	-1.01	-0.28	-1.10	-0.57
33087	90	58.7	-0.88	-1.40	-0.16	-1.23	0.57	-1.26	1.29	-0.92	1.65	0.05	-0.40	0.35	-0.76	0.83	-1.43	1.01	-1.82	0.24	-1.52	-0.74	-0.75	0.37	-1.15	0.99	-1.64	0.39	-1.18	-0.29	-1.28	-0.59
51272	99	62.0	-0.92	-1.46	0.00	-1.15	0.69	-1.19	1.37	-0.74	1.67	0.20	-0.34	0.28	-0.66	0.70	-1.57	0.95	-1.80	0.09	-1.46	-0.76	-0.82	0.34	-1.27	0.98	-1.75	0.37	-1.28	-0.26	-1.34	-0.56
92240	93	70.5	-1.13	-1.86	0.07	-1.39	0.72	-1.58	1.74	-1.03	2.03	0.19	-0.57	0.39	-1.02	1.07	-1.97	1.16	-2.31	0.08	-1.87	-0.97	-0.97	0.41	-1.49	1.21	-2.07	0.50	-1.53	-0.32	-1.60	-0.63
92949	63	84.5	-1.49	-2.47	-0.20	-1.79	0.81	-2.06	2.10	-1.32	2.63	0.12	-0.47	0.53	-1.22	1.53	-2.13	1.59	-2.76	0.24	-2.39	-1.05	-1.19	0.55	-1.90	1.48	-2.59	0.73	-1.84	-0.40	-1.92	-0.71
37304	101	87.5	-1.36	-2.00	-0.19	-1.62	0.63	-1.86	1.36	-1.62	2.35	-0.01	-0.52	0.57	-0.98	1.19	-1.87	1.28	-2.47	0.33	-2.05	-0.89	-1.07	0.44	-1.67	1.17	-2.28	0.56	-1.71	-0.33	-1.79	-0.65
53520	102	97.0	-1.45	-2.62	-0.18	-1.92	0.85	-2.20	2.24	-1.28	2.78	0.26	-0.90	0.70	-1.36	1.42	-2.32	1.50	-3.02	0.01	-2.43	-1.33	-1.29	0.65	-2.04	1.37	-2.79	0.73	-2.01	-0.41	-2.10	-0.74
70323	N.D.	103.0	-1.52	-2.40	0.00	-1.77	0.56	-2.10	1.68	-1.68	2.88	-0.02	-0.68	0.66	-1.29	1.38	-2.36	1.67	-3.09	0.12	-2.46	-1.12	-1.14	0.50	-1.88	1.24	-2.60	0.62	-1.88	-0.34	-1.96	-0.68
91915	109	112.0	-2.12	-3.77	0.00	-2.51	1.61	-2.99	2.97	-1.96	3.93	0.50	-1.23	1.18	-1.81	2.38	-3.21	2.31	-4.10	-0.12	-3.18	-1.90	-1.81	0.88	-2.93	1.72	-4.01	0.89	-2.95	-0.46	-3.07	-0.90
91892	136	117.0	-2.29	-3.87	-0.18	-2.76	1.31	-3.18	3.15	-2.08	4.26	0.23	-1.26	0.97	-2.42	2.57	-3.84	2.28	-4.57	-0.16	-3.47	-1.98	-1.92	0.82	-3.25	1.68	-4.53	0.85	-3.31	-0.46	-3.43	-0.90
53273	120	122.7	-1.96	-2.90	-0.94	-2.51	0.68	-2.80	2.39	-1.71	3.38	0.13	-0.67	0.82	-1.36	1.44	-2.68	1.69	-3.52	0.95	-3.22	-1.09	-1.60	0.57	-2.16	1.23	-2.80	0.62	-2.00	-0.33	-2.10	-0.65
37626	136	128.1	-2.21	-3.48	-0.21	-2.79	1.23	-3.02	3.00	-2.20	4.16	0.04	-0.97	0.76	-1.78	1.94	-3.83	1.96	-4.03	0.22	-3.41	-1.51	-1.78	0.68	-2.97	1.47	-4.12	0.73	-2.98	-0.40	-3.09	-0.73
37866	131	129.3	-1.96	-3.43	-0.30	-2.64	1.43	-2.84	2.83	-2.27	3.77	0.67	-0.80	0.59	-1.39	1.71	-2.35	2.20	-3.88	0.50	-3.32	-1.68	-1.62	0.50	-2.69	1.24	-3.73	0.65	-2.65	-0.37	-2.79	-0.75
53178	123	147.0	-3.76	-4.70	-0.59	-3.39	1.97	-3.67	3.90	-2.60	5.15	-1.29	-1.10	1.43	-1.95	2.51	-4.05	3.19	-5.86	-0.04	-4.90	-2.08	-2.48	0.91	-4.02	1.90	-5.49	1.10	-4.29	-0.53	-4.51	-1.03
91517	136	148.0	-3.06	-5.79	0.08	-3.84	1.79	-4.17	3.83	-2.92	5.58	0.55	-1.63	0.96	-2.14	2.63	-5.42	3.65	-6.99	-0.75	-5.47	-3.43	-2.78	0.84	-4.52	1.78	-6.22	0.97	-4.82	-0.60	-5.08	-1.20
53471	138	163.0	-1.96	-4.31	-0.08	-3.05	1.42	-3.44	3.12	-2.62	4.28	-0.34	-1.05	1.01	-2.59	2.61	-4.09	2.46	-4.48	-0.34	-3.61	-2.28	-2.01	0.76	-3.29	1.59	-4.54	0.82	-3.36	-0.45	-3.49	-0.91
53444	133	163.0	-2.58	-4.61	-0.36	-3.39	0.82	-3.39	2.50	-3.39	5.20	-0.34	-1.09	1.42	-2.23	2.33	-3.87	2.56	-4.86	0.42	-4.06	-2.09	-2.22	0.79	-3.62	1.63	-4.89	0.85	-3.68	-0.47	-3.80	-0.93
53503	N.D.	170.0	-3.94	-5.10	-0.69	-3.93	2.09	-4.96	5.38	-2.65	5.99	-0.02	-1.49	1.16	-3.10	2.95	-4.65	3.38	-6.49	-0.06	-5.59	-2.75	-2.99	0.95	-4.90	1.94	-6.76	1.19	-5.27	-0.65	-5.57	-1.29
53467	N.D.	185.0	-3.67	-5.85	-0.87	-4.32	2.71	-4.97	5.53	-2.80	6.11	0.29	-2.20	1.29	-3.33	3.07	-4.91	3.33	-6.39	2.23	-5.84	-2.61	-3.35	1.00	-5.40	2.09	-6.92	1.28	-5.43	-0.70	-5.75	-1.38
