# Supplementary material for: Morphogenetic development of trochlear groove and thigh muscles from embryo to fetus in humans
Source: PLoS One. 2026 Feb 2;21(2):e0339167. doi: 10.1371/journal.pone.0339167 (PMC12863510; doi:10.1371/journal.pone.0339167)

Supplemental File 1. The results of principal component analysis (PCA) and linear discriminant analysis (LDA) of Procrustes analysis

All samples

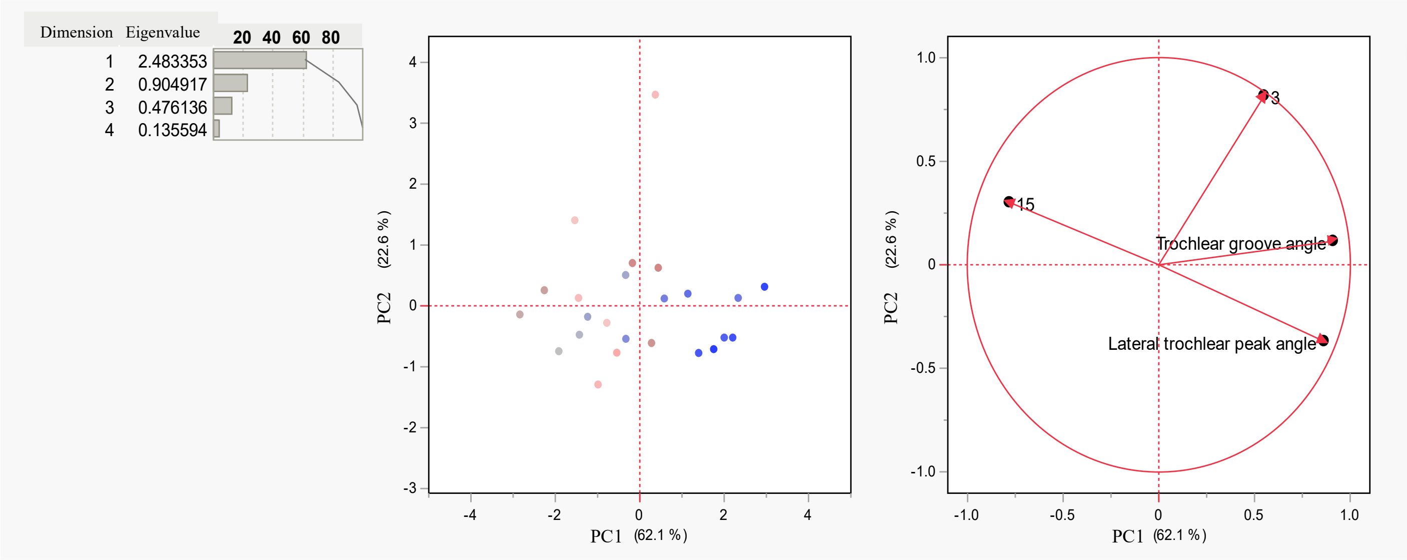

Loadings

Variable	PC1	PC2	PC3	PC4
Trochlear groove angle (2)	0.91062	0.11414	0.31146	-0.24646
Lateral trochlear peak angle (1)	0.86289	-0.36931	0.24954	0.23826
Medial trochlear peak (3)	0.55045	0.81553	-0.13231	0.12003
Medial point of the anterior part of the lateral condyle (15)	-0.77882	0.30068	0.54713	0.06065

Group1(CRL 21–80 mm, n = 9)

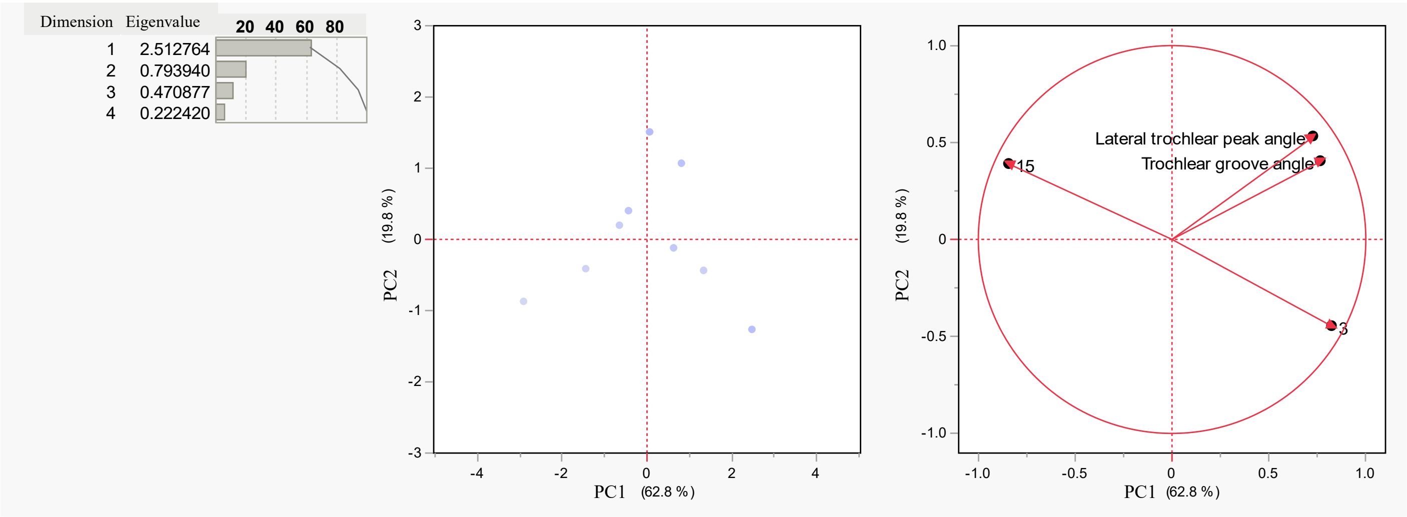

Loadings

Variable	PC1	PC2	PC3	PC4
Trochlear groove angle (2)	0.76823	0.40234	0.48303	-0.12092
Lateral trochlear peak angle (1)	0.73043	0.52997	-0.41092	0.12942
Medial trochlear peak (3)	0.82656	-0.44814	0.13965	0.31060
Medial point of the anterior part of the lateral condyle (15)	-0.84015	0.38777	0.22181	0.30753

Group2 (CRL 81–140 mm, n = 9)

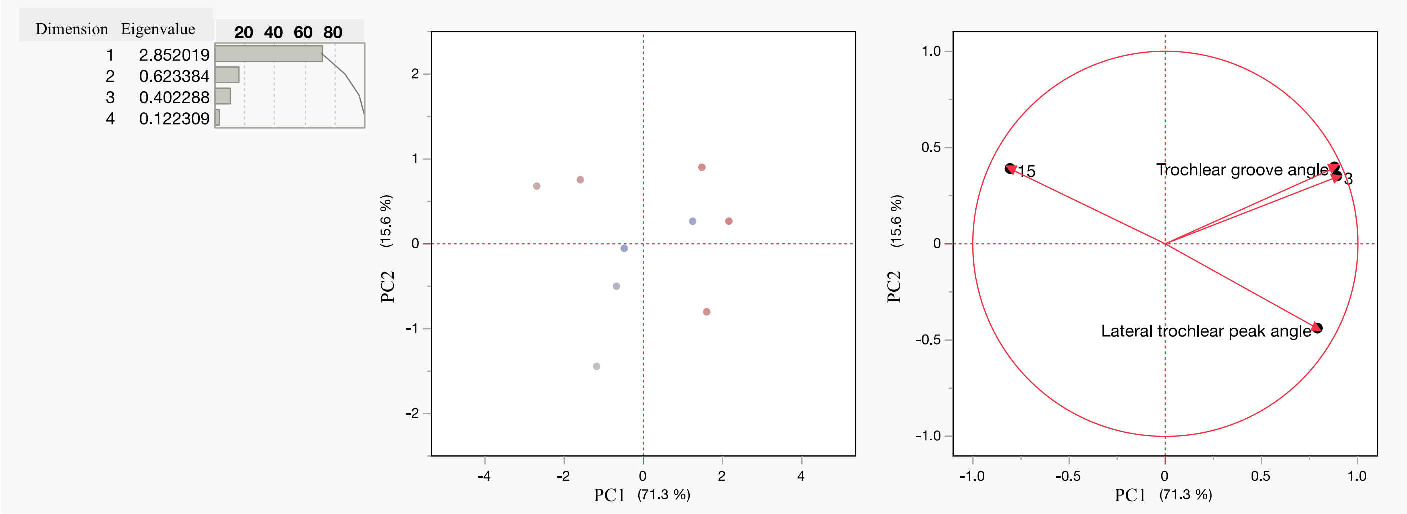

Loadings

Variable	PC1	PC2	PC3	PC4
Trochlear groove angle (2)	0.88152	0.39635	-0.10492	0.23413
Lateral trochlear peak angle (1)	0.79390	-0.44114	0.41179	0.07444
Medial trochlear peak (3)	0.89473	0.34856	0.14097	-0.24104
Medial point of the anterior part of the lateral condyle (15)	-0.80257	0.38755	0.44926	0.06208

Group3 (CRL 141–200 mm, n = 6)

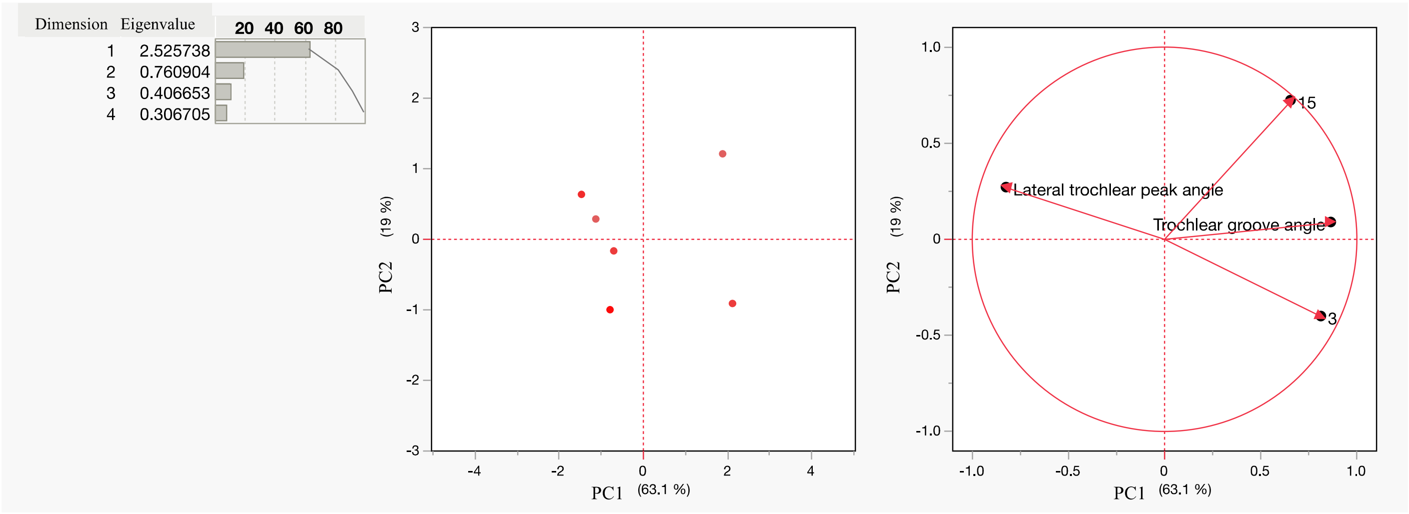

Loadings

Variable	PC1	PC2	PC3	PC4
Trochlear groove angle (2)	0.86762	0.08582	0.33477	-0.35750
Lateral trochlear peak angle (1)	-0.82081	0.26814	0.48771	0.12850
Medial trochlear peak (3)	0.81593	-0.40298	0.21586	0.35392
Medial point of the anterior part of the lateral condyle (15)	0.65841	0.72059	-0.10064	0.19269

The results of LDA

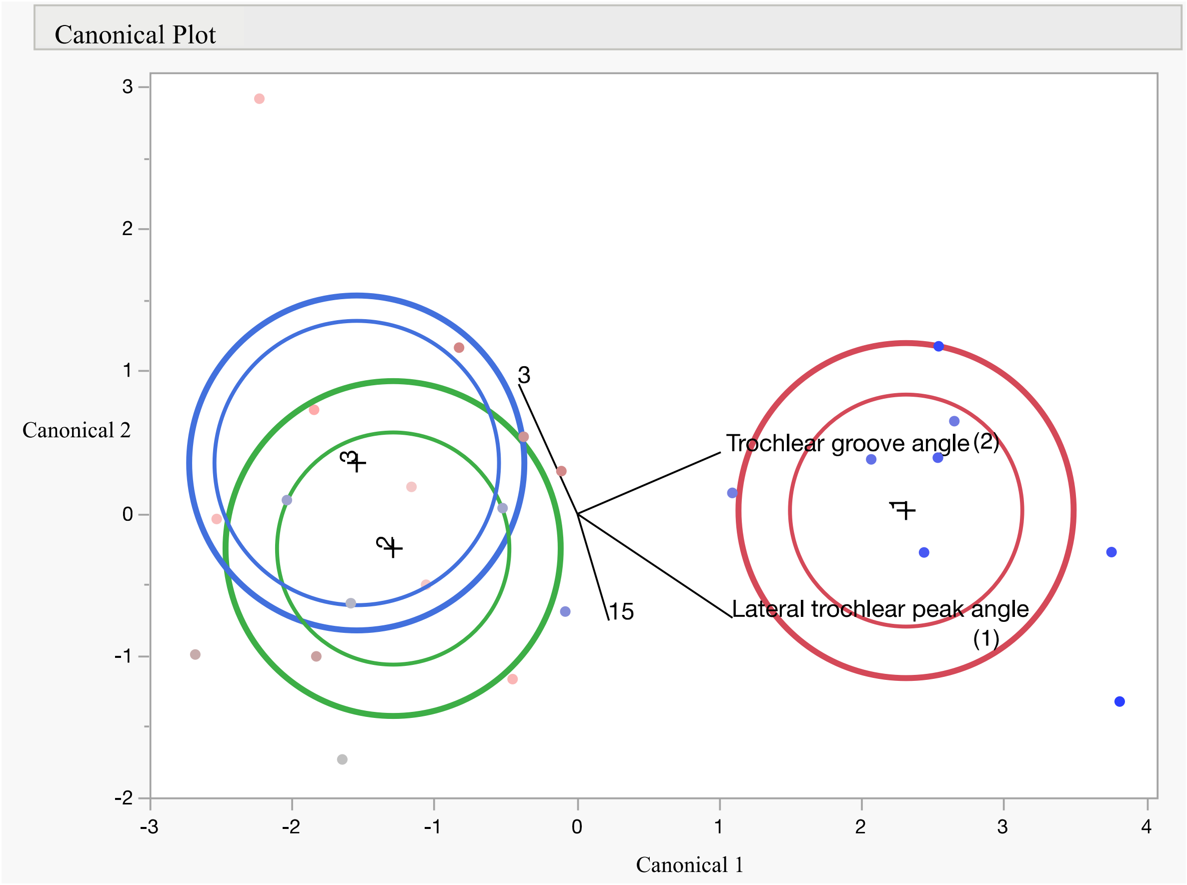

Supplement: S2 File — This file contains the results for the whole sample and each CRL group: score plots and loading tables from the PCA analysis, and canonical plots from the LDA analysis. (PDF) [file pone.0339167.s008.pdf]
