# Supplementary material for: Morphogenetic development of trochlear groove and thigh muscles from embryo to fetus in humans
Source: PLoS One. 2026 Feb 2;21(2):e0339167. doi: 10.1371/journal.pone.0339167 (PMC12863510; doi:10.1371/journal.pone.0339167)

Supplemental File 1. A scatter plot of trochlea Angle A or B and muscle motions

A scatter plot of trochlea Angle A and muscle motions

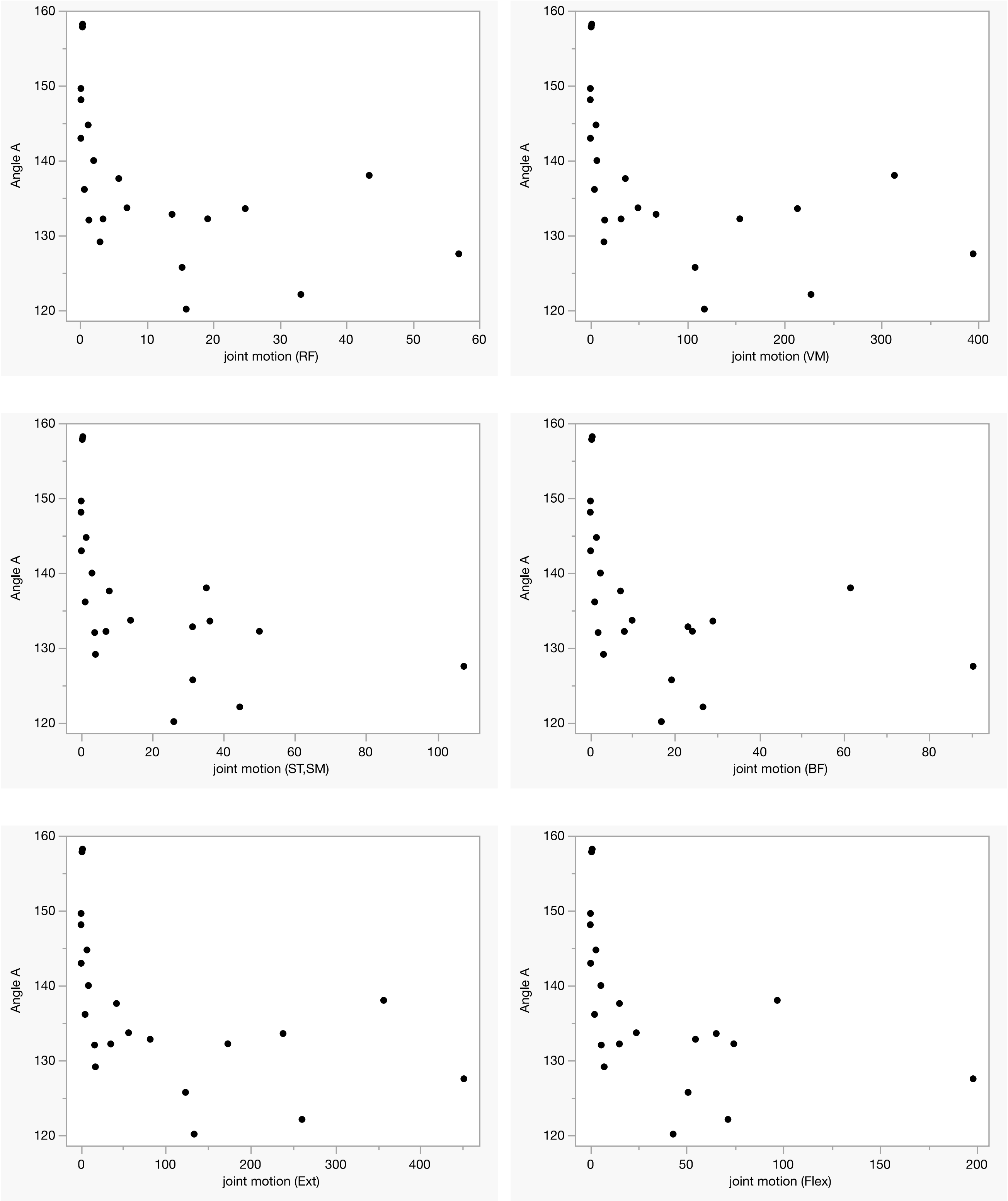

A scatter plot of trochlea Angle B and Muscle motions

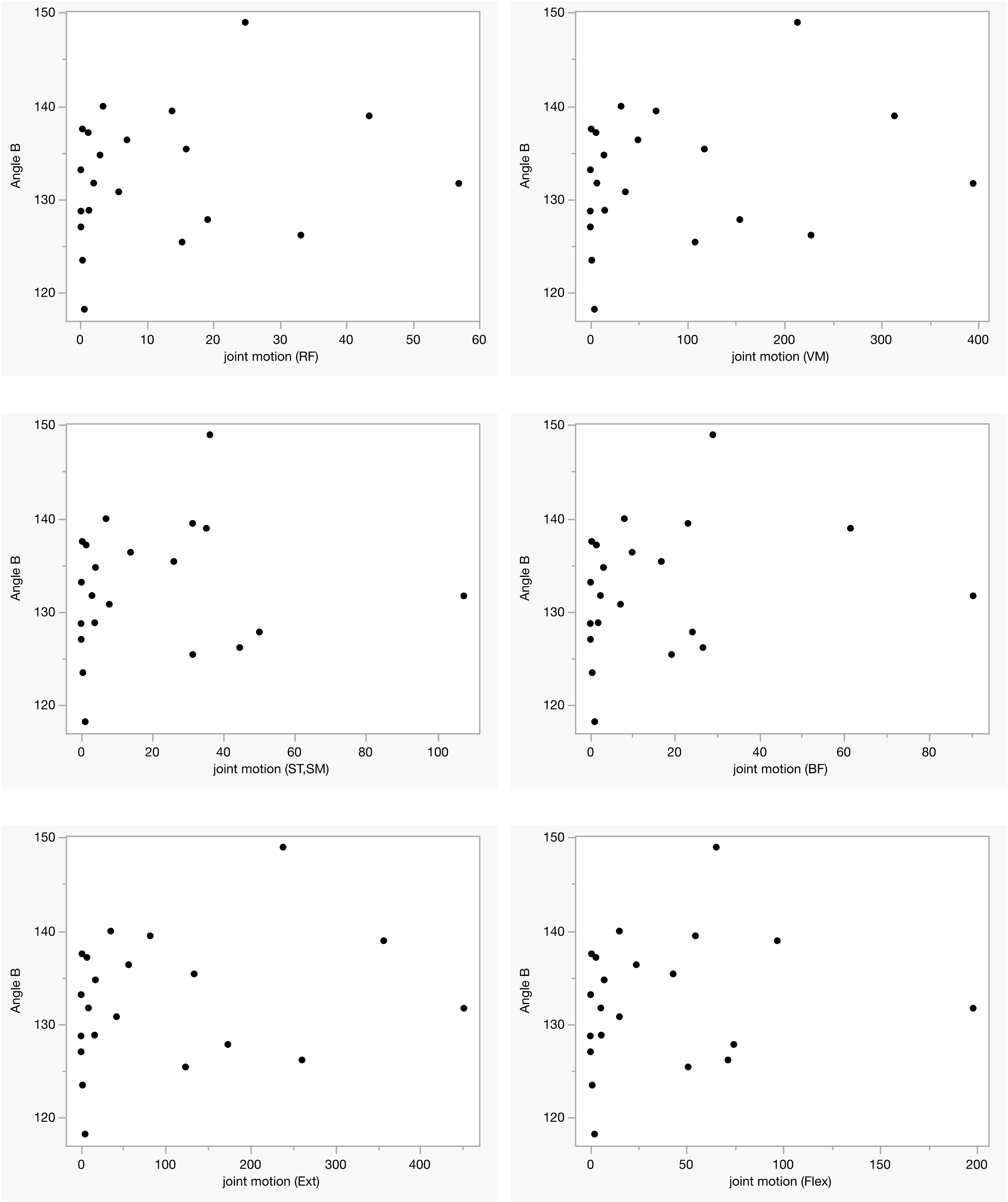

Supplement: S3 File — (PDF) [file pone.0339167.s009.pdf]
